# Family Socioecological Correlates of Lifestyle Patterns in Early Childhood: A Cross-Sectional Study from the EDEN Mother–Child Cohort

**DOI:** 10.3390/nu13113803

**Published:** 2021-10-26

**Authors:** Alexandra Descarpentrie, Cécilia Saldanha-Gomes, Claire Guivarch, Patricia Dargent-Molina, Blandine de Lauzon-Guillain, Sabine Plancoulaine, Marie-Aline Charles, Airu Chia, Mary Foong Fong Chong, Stéphanie Vandentorren, Barbara Heude, Jonathan Yoan Bernard, Sandrine Lioret

**Affiliations:** 1 Centre for Research in Epidemiology and StatisticS (CRESS), Inserm, INRAE, Université de Paris, F-75004 Paris, France; cecilia.saldanha-gomes@u-psud.fr (C.S.-G.); claire.guivarch@inserm.fr (C.G.); patricia.dargent@inserm.fr (P.D.-M.); blandine.delauzon@inserm.fr (B.d.L.-G.); sabine.plancoulaine@inserm.fr (S.P.); marie-aline.charles@inserm.fr (M.-A.C.); barbara.heude@inserm.fr (B.H.); jonathan.bernard@inserm.fr (J.Y.B.); sandrine.lioret@inserm.fr (S.L.); 2 Unité Elfe, Ined-Inserm-EFS, Aubervilliers, F-75020 Paris, France; 3 Saw Swee Hock School of Public Health, National University of Singapore and National University Health System, Singapore 117549, Singapore; airu-chia@nus.edu.sg (A.C.); mary_chong@nus.edu.sg (M.F.F.C.); 4 Singapore Institute for Clinical Sciences (SICS), Agency for Science, Technology and Research (ASTAR), Singapore 117609, Singapore; 5 Inserm, UMR1219, Vintage Team, Université Bordeaux, F-33000 Bordeaux, France; stephanie.vandentorren@santepubliquefrance.fr

**Keywords:** children, energy balance-related behaviors, lifestyle patterns, socioecological model, family

## Abstract

Energy balance-related behaviors (EBRBs), i.e., diet, sedentary behavior, physical activity, and sleep, combine into lifestyle patterns, which we aim to identify in French preschoolers and analyze their family correlates within the framework of a comprehensive socioecological model. Parental questionnaires provided information about family characteristics and children’s EBRBs for 978 5-year-olds of the EDEN cohort. We used principal component analysis to derive lifestyle patterns from EBRBs and hierarchical multivariable linear regressions to assess their associations with family socio-demographics, parent health/behaviors, and parent-child interactions. Analyses were stratified by sex. Of the three lifestyle patterns identified (unhealthy, healthy, and mixed), the mixed pattern differed the most between sexes. Lower parental education, suboptimal maternal diet, TV during meals, and later bedtime were associated with higher adherence to unhealthy patterns. Children cognitively stimulated at home and boys of mothers not employed adhered more to the healthy pattern. Older siblings (for girls) and higher engagement of parents in leisure-time physical activity (for boys) were related to greater adherence to mixed patterns. The identification of various correlates from multiple socioecological levels suggests that tackling the potentially synergistic effect of lifestyle patterns on health requires addressing processes relevant to the parent-child dimension and structural barriers parents may encounter.

## 1. Introduction

The prevalence of overweight in children and adolescents has increased fourfold since the past four decades worldwide [1], and reached a plateau in few high-income countries since the beginning of the century [2]. Still, overweight prevalence in these countries remains high and disproportionally affects socially disadvantaged children [3]. These children are more at risk of experiencing obesity later in life, along with its associated health consequences, well-being and socio-economic achievements [4]. 

Energy-dense and nutrient-poor diets, high levels of screen time, low levels of physical activity, and sleep deprivation, all known to contribute to energy imbalance, are established in early life stages and have been associated with higher risk of overweight in children [5]. Although often studied separately, energy balance-related behaviors (EBRBs) co-vary, as a result of common factors underlying the development of EBRBs or one behavior serving as a stimulus or coping strategy for another [6]. These combinations of behaviors could exert a synergistic effect on overweight [7,8,9,10]. Therefore, efforts to tackle childhood overweight are likely more efficient if early patterns of EBRBs are targeted, rather than isolated behaviors [6,11]. 

To account for this potential synergistic effect into the so-called “lifestyle patterns”, data-driven techniques have been used, including principal component analysis (PCA), cluster analysis and latent class analysis [7,8,9,10]. In children, lifestyle patterns were classified into three main types: unhealthy, healthy, or mixed (co-occurrence of both healthy and unhealthy EBRBs) [7,8,9,10]. Such patterns were shown to differ by sex, with boys scoring higher than girls on unhealthy patterns, as well as on those characterized by high physical activity and high screen sedentary behaviors (i.e., mixed patterns) [7,8,9,10]. However, only few studies have examined these patterns among children younger than 5 years. The recent and comprehensive review by D’Souza et al. pointed out that lifestyle pattern analyses rarely included sleep [9], despite its relations shown with eating and activity behaviors (i.e., physical activity and screen sedentary behaviors) [12,13]. It is also worth noting that published studies have so far examined a limited set of contextual factors in relation to these patterns, mainly focusing on a few dimensions of socioeconomic position (SEP), especially parental education level, occupation, household income and number of working hours [9]. However, the influence of the social context on EBRBs goes well beyond SEP and extends to a larger socio-ecological environment, the better knowledge of which is essential for developing effective health preventive actions.

The socio-ecological model as proposed by Bronfenbrenner represents nested spheres of the environment which influence child’s development, and is structured according to their proximity with the child’s experience [14]. Davison and Birch adapted this model to the issue of child overweight: the most proximal sphere influencing the child EBRBs corresponds to the familial context, whereas the community and societal contexts exert more distal roles [15]. Family, and particularly parents, play a pivotal role in shaping children’s EBRBs, especially for younger children. In view of the structural importance of background characteristics to address health issues in at-risk populations, the family sphere can also be disentangled into different levels of influence: those related to the family socio-demography, to parents’ health/behaviors, and to parent-child interactions [16]. Exploring not only maternal, but also paternal [17] and sibling, correlates of lifestyle patterns holistically may thus constitute important starting points for interventions to prevent suboptimal and promote optimal lifestyle patterns, shifting research from preventing to promoting health outcomes.

Our study therefore aimed to identify lifestyle patterns based on diet, screen time, outdoor play, walking, and sleep and to examine their family socio-ecological correlates. As suggested by previous reviews on lifestyle patterns [7,8,9,10], analyses were conducted separately for girls and boys. 

## 2. Materials and Methods

### 2.1. Study Design

We used data from the EDEN mother-child cohort, which was designed to assess pre- and postnatal determinants of child development and health [18]. In brief, between 2003 and 2006, 2002 pregnant women (<24-weeks’ gestation) aged 18–45 y were recruited in two university hospitals located in Nancy and Poitiers, France. Exclusion criteria were multiple pregnancies, known diabetes before pregnancy, not being able to speak or read French, and plan to move outside the region within 3 years.

### 2.2. Measurements

Data were collected from medical records (pregnancy, birth), clinical examinations (5 years), by trained interviewers (pregnancy and 5 years), child questionnaires completed by parents (2 and 5 years), and by mothers’, and fathers’ self-reported questionnaires at 5 years.

#### 2.2.1. EBRBs

The selection of EBRBs studied was guided by the literature [7,8,9,10] and their availability in the child questionnaire mailed to the parents when the children reached the age of 5 years.

**Diet:** Dietary intake was ascertained using a 27-item Food Frequency Questionnaire. This was a short version of the FFQ used by mothers during their pregnancies, which was validated in adults and adolescents [19]. In this version, the food classification was established based on similarities in food type and context of consumption and was set to be able to describe the patterns of the child diet. This questionnaire assessed the child’s dietary intake over a typical week (considering meals consumed at home and away from home), with seven response categories for each item, ranging from “never” to “several times per day”, then converted into weekly frequencies. Of the seventeen food items selected, fourteen were assembled into the following groups: “fish” (high fat fish and low fat fish), “dairy products” (milk and dairy yogurts/cottage cheese), “vegetables”(raw and cooked vegetables), “fruits” (fresh and stewed fruits), “sugar or artificially sweetened beverages (SASBs)” (fruit juice, soft drinks, and diet soft drinks), “sweet snacks & desserts” (biscuits, chocolate/candy, and ice creams/dairy puddings). The original food groups “processed meat”, “French fries,” and “crisps” items were retained as such.

**Screen, outdoor play, and walking times:** Parents indicated their child screen, outdoor play, and walking times on a typical weekday (excluding Wednesday), on Wednesday (an off-school day at that time in France), and on a typical weekend day, using an open-ended response. Appropriate weighting for these different types of days led to the three following behavioral variables: screen, outdoor play, and walking times (expressed in hours per day) [20]. Screen time was defined based on watching television or playing video or computer games. Outdoor play included time spent playing in a backyard, a park, or a playground, out of the school environment and was standardized by season [20]. Walking time included commuting to school, going to the nanny, and shopping with the parents.

**Sleep duration:** Parents reported their child in-bed and out-bed times, separately for weekdays and weekend days. Children’s sleep duration in the night (in hours per day) was calculated accordingly.

#### 2.2.2. Family Socio-Ecological Correlates

**Figure 1:**
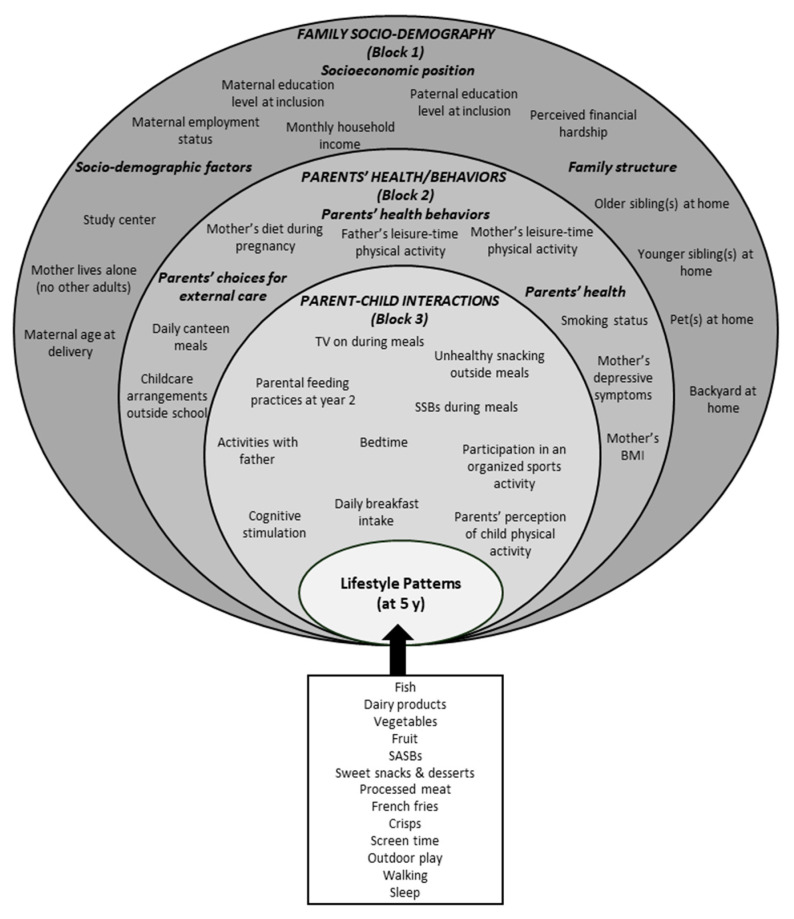
Conceptual framework of family contextual factors potentially associated with year-5 lifestyle patterns in the EDEN study. If not otherwise stated, factors were collected at year 5. BMI: Body mass index; SASBs: Sugar or artificially sweetened beverages; y: years.

The different contextual factors candidates for their possible association with lifestyle patterns were structured from the most distal to the most proximal within a three-nested-block framework (Figure 1), derived from both socio-ecological [15,21] and hierarchical [22] approaches, and were organized as follows: family socio-demography, parents health/behaviors, and parent-child interactions. These variables were selected on the basis of both the associations highlighted in the literature between contextual factors and each of the EBRBs under study [23], and their availability in the EDEN study. Given the cross-sectional approach, when available, we prioritized contextual factors collected at year 5. Otherwise, a few variables still thought valuable to consider before year 5 were selected because they are known to be relatively stable across early childhood. More details on the data used are provided in Additional Text S1 (“Family socio-ecological factors definition”).

### 2.3. Study Sample

**Figure 2:**
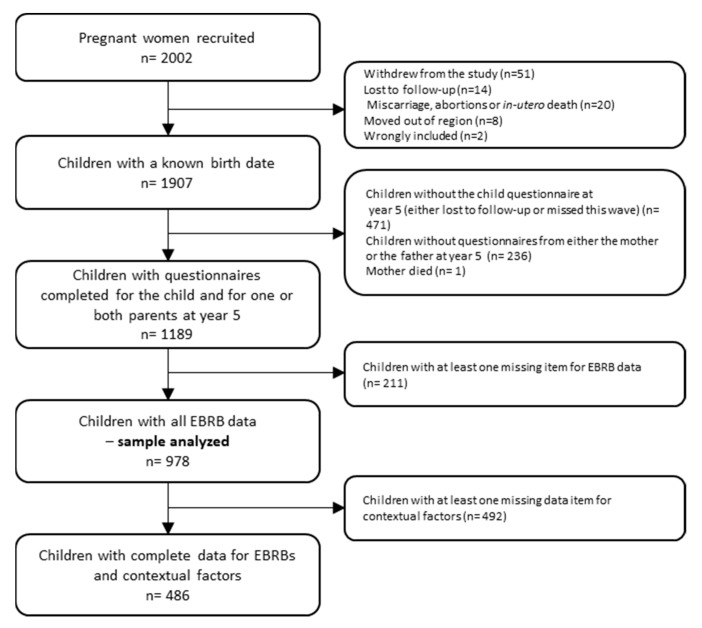
Flow chart of the population included in the study. The EDEN study. EBRBs: Energy balance-related behaviors.

Of the 2002 recruited mothers, 95 (4.7%) withdrew from the study during pregnancy, mainly for reasons of convenience, leaving 1907 children with a known birth date (Figure 2). Children were included in our analysis if their parents had completed the child questionnaire, as well as either the mother or the father self-reported questionnaire at year 5 (n = 1190). Complete data for all EBRBs was an additional criterion, which led to a study sample of 978.

### 2.4. Statistical Analysis

#### 2.4.1. Participant Characteristics

The study sample was compared to the EDEN population not included in this analysis (that is, live born children for whom any of the EBRBs was not available at year 5, n = 929) for parental demographic characteristics and SEP, as well as infant birth characteristics. Within the study population, we used *t*-tests to examine sex differences for continuous behavioral data, among children with all EBRBs available at year 5 (n = 978). After handling missing data, i.e., imputing contextual data and weighting respondents (see the Handling missing data section below), we described contextual variables according to sex. 

#### 2.4.2. Lifestyle Patterns 

PCA with Varimax rotation was conducted in girls and boys separately to synthesize the 13 standardized EBRB variables into fewer patterns. Since PCA is sensitive to outliers, their values (n = 1 for walking, n = 4 for screen viewing) were substituted with the maximum values of the remaining plausible distributions. The number of patterns was selected considering the analysis of the scree plot, eigenvalues > 0.1 and their interpretability [24]. Factor loadings represent the correlations of all standardized EBRBs with a given component (i.e., lifestyle pattern): we focused on factor loadings with an absolute value > 0.20 to characterize and label each pattern. For each participant and identified pattern a score was calculated: the higher the score, the greater the adherence to a given pattern (and vice versa).

#### 2.4.3. Hierarchical Linear Regression

First, for a given lifestyle pattern and the sake of parsimony, we selected contextual factors associated with *p* < 0.20 from the univariable analyses [25]. Hierarchical linear regression analyses were then conducted to examine the associations between contextual factors (independent variables) and lifestyle patterns (outcome variables), within the conceptual framework defined above (Figure 1) [22]. Variables were added per block from the most distal to the most proximal. In the multivariable analyses, each variable of a given block was interpreted within the first model in which it was included (i.e., model 1 for Block 1 variables; model 2 for Block 2 variables; model 3 for Block 3 variables), regardless of its performance in the subsequent model(s) [22,25,26]. This hierarchical approach was intended to ensure that intermediate variables did not affect the association of the distal factors with the outcome (i.e., lifestyle pattern) under study. 

#### 2.4.4. Handling Missing Data

Missing data were taken into account using a combined inverse-probability weighting and multiple imputation (IPW/MI) approach: IPW accounts for missing outcomes (i.e., lifestyle patterns) data whereas MI deals with missing data on contextual factors for these respondents (n = 978) [27]. As suggested by Seaman et al. [27] and Varshney et al. [28], we conducted a comparative sensitivity analysis to assess the impact of missing data. More information is available in Additional Text S2 (“Handling missing data—detailed procedure”).

All statistical analyses were performed using SAS^®^ 9.4 (SAS Institute Inc., Cary, NC, USA). The significance level was set at 0.05.

## 3. Results

### 3.1. Participants Characteristics

Compared to non-included children, children included in the present analysis were more likely to be born to an older and primiparous mother, who lived alone, with a higher SEP (education level, household income, perceived financial hardship), and was non-smoker prior to her pregnancy. However, there was no significant difference in mothers’ BMI before pregnancy between the two samples (Additional Table S4). The selected children’s, mothers’, and fathers’ characteristics are described in Table 1. Briefly, >60% of children’s mothers and fathers had at least a high school diploma; nearly 80% of mothers worked part- or full-time; 7.8% were living alone; and almost 40% were overweight obese. Boys had higher screen time [1.37 (0.80) vs. 1.26 (0.83) hours/day], higher intake of SASBs [6.90 (5.98) vs. 5.71 (4.92) time/week] and spent more time playing outdoors [0.07 (1.05) vs. −0.13 (0.94) SD per day] than girls (Table 2).

### 3.2. Lifestyle Patterns

Three lifestyle patterns were identified in girls as in boys, which accounted for 41% and 40% of the total variance, respectively (Table 2). They were defined based on their factor loadings as follows: “Discretionary Consumption, Low Vegetables, High Screen” in girls and “Discretionary Consumption, High Screen, Low Sleep” in boys (unhealthy); “Fish, Dairy products, Fruit & Vegetables, Low Screen” in both gender (healthy); “SASBs, High Screen, Outdoor Play, Walking, Low Sleep” in girls and “Dairy products, High Screen, Outdoor Play, Walking, High Sleep” in boys (mixed). For the sake of fluid writing, we will use the patterns abbreviation thereafter, i.e., unhealthy, healthy, and mixed

### 3.3. Hierarchical Linear Regression

The results from the univariable and hierarchical multivariable linear regressions are presented in Additional Tables S5–S7 and Table 3, Table 4 and Table 5, respectively. Thirteen contextual factors were related to, at least, one lifestyle pattern in girls, and eighteen in boys, nine of which were common to both genders (Table 3, Table 4 and Table 5). 

Briefly, for the unhealthy patterns (Table 3), from the most distal to the most proximal levels, associations were observed with maternal education level (inverse association for boys); paternal education level (inverse for all); pets at home (higher scores for children with at least a dog vs. no pets at home), backyard at home (inverse for girls); mother’s adherence to the Western (positive for all), and Healthy (positive for boys) dietary patterns; father’s leisure-time physical activity (inverse for boys); TV on during meals (positive for all), unhealthy snacking outside meals (positive for all), SASBs during meals (positive for boys), and bedtime (higher scores for children with later bedtime). 

For the healthy patterns (Table 4), associations were observed for maternal working status (higher scores for boys of not employed mothers vs. employed full-time); mother lives alone (positive for girls); mother’s adherence to the Healthy (positive for all) dietary patterns; mother’s depression symptoms (inverse for girls); the “restriction for health” feeding practice (positive for boys); unhealthy snacking outside meals (inverse for all); parental perception of child physical activity (lower scores for boys perceived more active than other children vs. less or as active as other children); participation in organized sports activity (positive for boys); bedtime (lower scores for boys with later bedtime); and cognitive stimulation (positive for all). 

For the mixed patterns (Table 5), associations were observed for maternal education level (inverse for all); paternal education level (inverse for boys); study center (lower scores in Nancy vs. Poitiers for boys); older siblings at home (positive for girls); pets at home (higher scores for children with one dog vs. no pets at home); father’s and mother’s leisure-time physical activity (both positive for boys); childcare arrangements outside school (lower scores in boys for those cared for in structured settings vs. by a family member); daily breakfast intake (inverse for girls); and bedtime (higher scores for girls with later bedtime and lower scores for boys with later bedtime).

### 3.4. Sensitivity Analyses

We found consistent direction of associations with the three different approaches used, although differences were observed in some effect sizes, notably in the complete cases analysis, along with a lower statistical power (data not shown, available on request).

## 4. Discussion

To our knowledge, this is the first study to comprehensively explore multiple socio-ecological determinants of lifestyle patterns including not only diet, screen sedentary behavior, and physical activity, but also sleep, in preschool girls and boys separately. We identified three distinct lifestyle patterns in 5 years-old children, in both sexes, which can be classified as unhealthy, healthy, and mixed. The mixed pattern was the one that differed the most between sexes, while the healthy pattern was the most similar. Whereas we confirmed their relation to SEP (parents’ education level), we underlined new associations with other distal (e.g., mother lives alone, older siblings at home), intermediate (e.g., mothers’ depression symptoms and parents’ lifestyle behaviors), and proximal (e.g., restrictive feeding practices, meal habits, cognitive stimulation, and bedtimes) family factors. Given the relatively large number of socio-ecological correlates identified, for the sake of being concise, we will thereafter focus on discussing associations we consider novel or of particular interest as prevention levers.

### 4.1. Lifestyle Patterns

**Unhealthy.** In both girls and boys, we confirmed a pattern commonly identified in the literature across ages and countries, characterized by energy-dense and nutrients-poor consumption along with high screen time [7,8,9,10]. In the present study, “low sleep” was an additional feature of the boys’ unhealthy pattern. According to D’Souza et al., in the scarce lifestyle pattern studies that have accounted for sleep along with other EBRBs, suboptimal sleep was often found to co-occur with high intake of discretionary food and beverages or high screen time items [9]. 

**Healthy.** The healthy lifestyle pattern observed in girls and boys was defined by high fish, dairy products, fruit, and vegetables intake, as well as low screen time, which is not the predominant combination reported in the literature [7,8,9,10]. Indeed, unlike our findings, scholars have pointed out that healthy lifestyle patterns are most often characterized by a high level of physical activity and either a low level of sedentary behavior or a balanced diet (or, to a lesser extent, the three of them). 

**Mixed.** Consistent with other studies [7,8,9], it is the combination of physical activity and screen times (both at relatively high levels) that contributed to the mixed profile of this pattern. However, in girls this mixed activity (high screen time / high outdoor play time) pattern combined with rather unhealthy features (i.e., higher level of SASBs consumption and less sleep time), whereas in boys, it combined with rather healthy behaviors (i.e., higher levels of dairy products consumption and longer sleep time). Although these findings are not easily comparable with the literature (due to the scarcity of studies which have considered sleep along with other EBRBs), mixed patterns comprising healthy dietary intake, high screen behavior and low physical activity, have more commonly been reported in girls, whereas those combining suboptimal dietary intake, high screen and physical activity, have most often been described in boys [9]. 

### 4.2. Socio-Ecological Correlates of Lifestyle Patterns

#### 4.2.1. Family Socio-Demography

Consistent with other lifestyle patterns’ studies, we replicated the finding that children born to mothers and fathers with lower educational backgrounds were more likely to adhere to suboptimal lifestyle patterns [7,8,9], either unhealthy or mixed. Children from parents with low educational background tend to live in socio-economically disadvantaged neighborhoods, with lower access to healthy food outlets (i.e., greater concentration of fast food restaurants as well as corner stores) [29], and possibly less safe and much more noise that could impair optimal sleep [30]. Such educational differences could also translate into lower health literacy experienced by parents, impairing their reception and active engagement with health promotion messages, whether the latter concern the promotion of healthy foods, low exposure to screen, but also sleep. The mixed patterns were also characterized by high levels of outdoor play, which could reflect less supervised activities. It was suggested that children with lower SEP were more likely to be granted greater independence than those with high SEP, owing to differences in parenting styles and social norms; it is also likely that these children live in areas where there are higher levels of social cohesion and thus parents feel their children are “safe” playing out unsupervised [31]. 

Social and economic disadvantage is particularly important for understanding lower levels of well-being among children cared after by single mothers [32]. However, in the present study, girls from mothers living alone had higher scores on the healthy lifestyle pattern. This finding seems to refute, at least in this rather educated sample, the hypothesis that budget constraints but also emotional problems, such as those more likely experienced by single parents, may result in more energy-dense and nutrient-poor diets [33]. Consistent with our result, Kiefte-de Jong et al. found that children of mothers living alone were more likely to adhere to a ‘Health conscious’ dietary pattern [34]. They believe that this might relate to other socio-demographic factors such as which person is responsible for meals preparation: it was demonstrated that cooking performed by a person other than the mother was negatively correlated with a ‘Healthy’ dietary pattern in 3-year-olds [35]. 

Irrespective of their living arrangements, another type of barrier mothers may experience is time constraints, and this might affect children’s lifestyle patterns, as suggested by the inverse relation underlined in this study between maternal employment and the boys’ healthy lifestyle pattern. Less time available to dedicate to a child may not only affect their diet but also their exposure to screen and opportunities for engaging in physical activities [36,37]. One mitigating factor may be that fathers or partners take more responsibility for shopping, preparing meals, and other child-rearing tasks when mothers work [38]. However, the fathers’/partners’ employment status at age 5 was not available, making it impossible to examine such potential influence on children lifestyle patterns further. 

Older siblings form an important dimension of a child’s experience of family life and constitute potential playmates [39]: such peer interactions could increase children physical activity. This hypothesis is supported by the current study, that among girls for whom having older sibling(s) was positively associated with mixed lifestyle pattern, characterized, among other EBRBs, by a high level of outdoor play and walking. Peer modelling is another likely mechanism: the social learning theory posits that younger siblings emulate the behaviors of the older siblings, which suggests a positive relation between younger and older siblings’ behaviors, such as physical activity but also food intake and screen time [40]: of note, SASBs and screen time were other key dimensions of mixed patterns in girls. Modeling these two EBRBs is then possible as well [41].

#### 4.2.2. Parents’ Health/Behaviors

Parents are also key role models for their child aged 5 years. We found that boys whose mothers and fathers engaged in more than 2 h of leisure sports per week scored higher on the mixed “Dairy products, High Screen, Outdoor Play, Walking, High Sleep” pattern compared to those whose parents did not engaged in any leisure sports. Active parents are also more likely to provide instrumental support, encouragement, and appraisal for sport and outdoor activities to their children [42]. Previous studies suggested that parents support was more pronounced for boys than for girls, which could explain why this association was observed in boys only [43,44]. At year 5, considering that maternal diet is rather consistent over pregnancy and the first post-partum years [45,46], parents modelling and support seems to hold true for diet as well. In both girls and boys, the more the mother adhered to the Western or Healthy dietary patterns, the higher the child scored on unhealthy and healthy patterns, respectively. However, the positive association between maternal Healthy dietary pattern and boys’ unhealthy pattern was counter-intuitive, with no straightforward explanation to suggest. 

As suggested by the inverse relation between mothers’ depression symptoms and girls’ healthy pattern, the mental health status of the mother might influence the child’s dietary intake. Arising from this health status, limited abilities to appropriately respond when feeding their children might include less motivation to shop for healthy groceries or cook [47]. On top of that, depressed mothers may use television as a coping solution [48]. Such possible influence could be equally related to the presence of paternal depressive symptoms [49].

#### 4.2.3. Parent-Child Interactions

At this young age, parents are still the main providers of foods and thus control their availability and accessibility. Our findings suggested that boys in the third tertile of the “restriction for health” parental feeding practice at 2 year had higher scores on the healthy pattern compared to their other counterparts. In other studies, restriction of food was associated with both excessive and reduced discretionary food intake [50]. These inconsistent results were suggested to reflect the contradictory nature of controlling food-related parenting practices, with some forms of control having beneficial effects whilst others may be detrimental [51,52]. Furthermore, in our study, children often provided with SASBs and exposed to TV during meals, which are likely indicators of a permissive parenting style or of family suboptimal shared habits, adhered more to the unhealthy pattern. This finding resonates with research showing that these unfavorable mealtime habits were useful to distinguish clusters of 2 and 5 years-old children with high energy-dense nutrients-poor food/drink intakes and high levels of TV exposure [20].

Increased parental engagement in their child’s stimulation activities, partly likely due to their knowledge of the potential detrimental effect of screens, may, on the contrary, result in less screen time exposure because more free time is shared with parents in non-screen related activities [53]. In our study we actually found that the HOME score (reflective of language stimulation, academic stimulation, and variety of experimentations) was positively associated with girls’ and boys’ healthy lifestyle patterns. Likewise, the review by Duch et al. [54] revealed that cognitive stimulation in the home environment was inversely associated with screen media for young children. More specifically, fathers’ engagement in stimulation activities might play a key role on child screen time: at the margin of significance, the more the father adhered to the "Every day care without physical leisure time" pattern (characterized by high weekly frequencies of bathing and reading for their child and low weekly frequencies of playing as well as walking with them), the less they scored on the mixed pattern, defined by a high level of screen time.

Bedtime routines are important for children’s sleep duration [55]. In our study, bedtime on the whole was associated in the expected directions with all lifestyle patterns: for patterns directly characterized by sleep time, the later the bedtime the lesser the sleep time (and vice versa). For patterns not directly characterized by sleep but by screen times, in accordance with the displacement hypothesis, the greater the screen time, the later the bedtime, and therefore, the lesser the sleep time (and vice versa).

### 4.3. Public Health Implications

Acknowledging the inability to tease out cause and effect from this cross-sectional study, our findings still indicate that distal and not easily modifiable predictors identified in this study (e.g., parents’ education), are worth considering to adapt the content of interventions to the most at-risk families experiencing specific vulnerabilities. In fact, general programs are often based on the sole information on healthy vs. unhealthy EBRBs and were shown to favor subgroups with more resources (such as specific knowledge, skills, income, and time) and self-efficacy to achieve changes, thus increasing social inequalities in behaviors and health [56,57]. Therefore, by providing parents with the needed resources but also with psycho-social support [58], the underlined associations of more proximal and modifiable contextual factors with lifestyle patterns may constitute precious information regarding potential practical daily tips, e.g.: promoting favorable meal habits, improving sleep routines, modeling healthy diet and sports activities, and promoting non-screen related activities. Also, such family-based interventions are susceptible to be more effective if they involve not only mothers, but also fathers and siblings. 

### 4.4. Limitations and Strengths

Our study must be interpreted in light of some limitations. First, the EDEN population has a relatively higher SEP than the general population. Caution is needed when attempting to generalize our study findings to the whole French population. However, we identified the unhealthy pattern commonly encountered in the literature in different populations, as well as its usual inverse association with SEP. Second, attrition and missing data were proportionately higher in children of low SEP families (Additional Table S4) and this selection bias limits further our ability to generalize our findings. Nonetheless, we minimized possible biases by using a combination of MI and IPW [27], and our findings were very consistent across the sensitivity analyses undertaken. Third, our study relies on parental report for EBRBs and contextual data that could have led to measurement error. This includes potential differential bias such as social desirability [59] and impaired precision. Some have suggested the use of accelerometry in studies examining combination of movement behaviors in children [60], given that these objective measurements undeniably provide more precise and complete data on the intensity and duration of total physical activity. However, they do not distinguish between different activity types and the various contexts in which they take place [60], which are valuable knowledge to be translated into preventive interventions. Finally, we included as many paternal factors as possible in the models provided they were relevant to our socioecological framework and available in the EDEN cohort (i.e., education level, physical activity and interactions with their child). However, we acknowledge that they are relatively limited as compared to the number of maternal factors we accounted for. 

This study has also strengths worth mentioning. First, while a number of previous studies have examined children’s lifestyle patterns [9], we are not aware of other studies examining the latter including sleep, in young girls and boys separately as well as their contextual correlates using such a holistic approach. Since family constitutes a cornerstone in child development, especially during infancy and early childhood, we have limited the socio-ecological approach to the family sphere, but the latter was addressed in a comprehensive way, which is novel. We cannot, however, ascertain whether more distal correlates, such as those related to the built environment (indefinitely approximated by the study center) or societal conditions, may have been important in shaping children’s lifestyle patterns. Finally, although the cross-sectional design and the exploratory approach did not allow causal inferences, the hierarchical approach prevented over-adjustment for potentially mediating variables and allowed the generation of new hypotheses to be addressed in future inferential and intervention studies.

## 5. Conclusions

Our findings highlighted that the co-variance of multiple EBRBs, including sleep, are observed in children as young as 5 years, and partly different between girls and boys. These findings add novel evidence into the literature on lifestyle patterns by the identification of valuable intervention entering point at different family socio-ecological levels. Combined with previous studies’ findings, our results may inform future etiological analyses and interventional studies. Indeed, not only suboptimal behaviors were found to cluster and track from toddlerhood [20,61], but were also prospectively associated with later adiposity [20,62], thus reinforcing the importance of intervening in early life. Tackling EBRBs individually may fail to reflect the complexity of children’s lives and how they experience related behaviors. Despite their shared variance and potential synergy, and depending on the child context of living, changing one EBRB may be easier than another one [63,64]: social inequalities and inequities exist in the ability and opportunities to successfully apply appropriate preventive messages to adopt optimal EBRBs. Therefore, in addition to tackling multiple EBRBs, multi-level interventions to facilitate behaviors change should ensure that their socio-ecological correlates, both distal (i.e., barriers or facilitators) or proximal (i.e., practical tips), are considered.

## Figures and Tables

**Table 1:** Population characteristics at year 5, according to the child’s sex. The EDEN study.

	Weighted Mean or % (95% CI)		
	All (n = 978)	Girls n = 459	Boys n = 519
***BLOCK 1: FAMILY SOCIO-DEMOGRAPHY***			
**Maternal education level at inclusion**			
<High school diploma	29.3 (25.8; 32.7)	27.9 (22.8; 32.9)	30.5 (25.8; 35.2)
High school diploma to 2-year university degree	39.3 (36.1; 42.5)	40.9 (36.1; 45.7)	37.9 (33.5; 42.3)
≥3-year university degree	31.4 (28.6; 34.3)	31.2 (27.0; 35.4)	31.6 (27.7; 35.6)
**Paternal education level at inclusion**			
<High school diploma	37.2 (33.7; 40.7)	36.2 (31.0; 41.4)	38.1 (33.4; 42.9)
High school diploma to 2-year university degree	38.9 (35.6; 42.3)	38.3 (33.4; 43.3)	39.5 (34.9; 44.0)
≥3-year university degree	23.9 (21.2; 26.5)	25.5 (21.4; 29.5)	22.4 (18.9; 25.9)
**Monthly household income**			
<2300 EUR	27.8 (24.5; 31)	25.1 (20.4; 29.8)	30.2 (25.6; 34.7)
2301 EUR to 3800 EUR	45.7 (42.4; 49.1)	47.4 (42.5; 52.2)	44.3 (39.7; 48.9)
>3800 EUR	26.5 (23.7; 29.2)	27.5 (23.4; 31.7)	25.5 (21.8; 29.2)
**Perceived financial hardship**			
At least one	18.1 (15.2; 20.9)	17.4 (13.3; 21.5)	18.6 (14.7; 22.6)
**Maternal employment status**			
Not employed	21.7 (18.8; 24.7)	20.7 (16.6; 24.9)	22.7 (18.4; 26.9)
Employed part-time	29.1 (26.1; 32.2)	31.3 (26.6; 35.9)	27.2 (23.2; 31.2)
Employed full-time	49.2 (45.8; 52.5)	48.0 (43.1; 52.9)	50.1 (45.6; 54.8)
**Study center**			
Nancy	50.1 (46.7; 53.4)	52.8 (47.9; 57.7)	47.6 (43.0; 52.2)
**Maternal age at delivery**			
<27 y	26.8 (23.6; 29.9)	26.0 (21.4; 30.6)	27.5 (23.1; 31.9)
27 to 33 y	48.0 (44.6; 51.3)	49.4 (44.5; 54.3)	46.7 (42.1; 51.3)
>33 y	25.2 (22.5; 28.0)	24.6 (20.7; 28.6)	25.8 (22.1; 29.6)
**Mother lives alone (no other adult)**			
Yes	7.8 (5.8; 9.8)	7.8 (4.8; 10.8)	7.8 (5.1; 10.4)
**Younger siblings at home**			
At least one	46.3 (43.0; 49.7)	46.5 (41.6; 51.5)	46.1 (41.5; 50.7)
**Older siblings at home**			
At least one	55.5 (52.3; 58.8)	55.0 (50.2; 59.8)	56.0 (51.5; 60.5)
**Pets at home**			
At least one dog	29.7 (26.6; 32.9)	29.1 (24.5; 33.7)	30.3 (26.0; 34.6)
No dog but other animals	31.7 (28.6; 34.9)	32.8 (28.1; 37.4)	30.8 (26.6; 35.0)
No pets	38.6 (35.3; 41.7)	38.1 (33.5; 42.8)	38.9 (34.4; 43.3)
**Backyard at home**			
Yes	89.8 (87.4; 92.2)	88.6 (84.9; 92.3)	90.9 (88.0; 93.8)
***BLOCK 2: PARENTS’ HEALTH/BEHAVIORS***			
**Mother’s diet during pregnancy (PCA scores)**			
Healthy	−0.03 (−0.10; 0.03)	−0.04 (−0.14; 0.06)	−0.03 (−0.11; 0.06)
Western	−0.02 (−0.10; 0.05)	−0.03 (−0.13; 0.07)	−0.02 (−0.13; 0.0)
**Mother’s leisure-time physical activity**			
0 h/week	58.4 (55.2; 61.7)	61.0 (56.3; 65.7)	56.1 (51.6; 60.6)
>0 to 2 h/week	27.5 (24.6; 30.4)	27.1 (23.0; 31.3)	27.8 (23.9; 31.7)
>2 h/week	14.1 (11.8; 16.3)	11.8 (8.9; 14.8)	16.1 (12.8; 19.4)
**Father’s leisure-time physical activity**			
0 h/week	50.9 (47.5; 54.4)	54.1 (49.1; 59.0)	48.1 (43.3; 52.9)
>0 to 2 h/week	18.4 (15.7; 21.1)	17.5 (13.8; 21.3)	19.1 (15.4; 22.9)
>2 h/week	30.7 (27.6; 33.9)	28.4 (24.0; 32.9)	32.8 (28.4; 37.2)
**Mother’s BMI**			
>30 kg/m^2^	15.8 (13.3; 18.3)	16.8 (13.1; 20.6)	14.9 (11.6; 18.3)
25 to 30 kg/m^2^	23.9 (20.9; 26.8)	24.6 (20.2; 29.0)	23.2 (19.2; 27.2)
<25 kg/m^2^	60.3 (57.0; 63.6)	58.6 (53.7; 63.5)	61.8 (57.3; 66.4)
**Mother’s depression symptoms (CES-D score)**	9.57 (9.02; 10.13)	9.84 (9.00; 10.69)	9.33 (8.59; 10.06)
**Smoking status**			
At least one parent smokes	47.5 (44.1; 50.9)	48.3 (43.3; 53.3)	46.8 (42.1; 51.5)
**Childcare arrangements outside school**			
Outside school hours care services	36.1 (32.9; 39.2)	34.0 (29.5; 38.5)	37.9 (33.6; 42.3)
Neighbor or employee	23.4 (20.6; 26.2)	28.1 (23.7; 32.5)	19.2 (15.8; 22.7)
Mother, father (parents) or family	40.5 (37.2; 43.9)	37.9 (33.0; 42.8)	42.8 (38.2; 47.4)
**Daily canteen meals**			
Yes	53.7 (50.3; 57.1)	55.3 (50.3; 60.4)	52.3 (47.7; 56.9)
***BLOCK 3: PARENT-CHILD INTERACTIONS***			
**Parental feeding practices at year 2**			
**Child control (Lack of parental control)**			
T3 (tertile range: 2.66 to 5)	28.9 (25.8; 32.1)	26.8 (22.4; 31.4)	30.8 (26.4; 35.3)
**Food as reward**			
T3 (tertile range: 1.66 to 4)	32.0 (28.7; 35.4)	34.3 (29.4; 39.3)	30.0 (25.4; 34.5)
**Food restrictions for health**			
T3 (tertile range: 4.25 to 5)	37.9 (34.4; 41.4)	36.8 (31.6; 41.9)	38.8 (34.0; 43.6)
**Pressure to eat**			
T3 (tertile range: 3 to 4.66)	33.3 (29.9; 36.8)	32.9 (28.0; 37.7)	33.8 (29.1; 38.4)
**Daily breakfast intake**			
Yes	91.9 (90.0; 93.8)	91.3 (88.2; 94.3)	92.5 (90.1; 94.8)
**TV on during meals**			
Often or always	33.0 (29.8; 36.3)	31.9 (27.1; 36.6)	34.2 (29.7; 38.6)
Sometimes	32.7 (29.5; 35.8)	33.2 (28.6; 37.7)	32.2 (27.9; 36.5)
Never	34.3 (31.2; 37.3)	35.0 (30.4; 39.5)	33.6 (29.4; 37.8)
**Unhealthy snacking outside meals**			
Often or always	18.8 (15.9; 21.6)	18.0 (13.9; 22.0)	19.5 (15.6; 23.4)
Sometimes	58.0 (54.7; 61.4)	56.6 (51.7; 61.5)	59.3 (54.8; 63.9)
Never	23.2 (20.5; 25.9)	25.5 (21.3; 29.7)	21.2 (17.7; 24.7)
**SASBs during meals**			
Yes	20.1 (17.2; 23.0)	18.3 (14.1; 22.4)	21.7 (17.8; 25.7)
**Parents’ perception of child physical activity**			
More active than other children	17.9 (15.3; 20.6)	12.1 (8.7; 15.5)	23.1 (19.2; 27.0)
**Participation in an organized sports activity**			
Yes	55.4 (52; 58.7)	60.2 (55.3; 65.1)	51.0 (46.4; 55.6)
**Bedtime (hours and minutes)**	20 h 42 min (20 h 40 min; 20 h 44 min)	20 h 42 min (20 h 39 min; 20 h 44 min)	20 h 43 min (20 h 40; 20 h 45 min)
**Cognitive stimulation (HOME score)**	17.10 (16.92; 17.26)	17.10 (16.82; 17.31)	17.12 (16.89; 17.34)
**Activity with the father (PCA scores)**			
Everyday care with leisure time	0.00 (−0.08; 0.07)	−0.10 (−0.17; 0.05)	0.04 (−0.05; 0.15)
Everyday care without active leisure time	−0.07 (−0.15; 0.01)	−0.01 (−0.12; 0.10)	−0.12 (−0.24; −0.01)

If not otherwise stated, variables were collected at year 5. “Child control” and “Food restrictions for health” total scores range from 1 to 5, “Food as reward” from 1 to 4; “Pressure to eat” from 1 to 4.66. BMI: Body mass index; CES-D: Centre for Epidemiologic Studies-Depression; CI: Confidence interval; HOME: Home observation measurement of the environment; PCA: Principal component analysis; T: Tertile; y: years.

**Table 2:** EBRB distribution and PCA factor loadings for lifestyle patterns at year 5. The EDEN study.

	Girls (n = 459)				Boys (n = 519)				
	EBRB Distribution	PCA Factor Loadings			EBRB Distribution	PCA Factor Loadings			
	Mean (SD)	Unhealthy	Healthy	Mixed	Mean (SD)	Unhealthy	Healthy	Mixed	*p*
Fish (times/week)	1.94 (1.47)	0.16	**0.60**	−0.01	1.96 (1.41)	0.00	**0.59**	0.04	0.84
Dairy products(times/week)	15.90 (6.77)	0.03	**0.37**	−0.04	16.26 (6.85)	0.11	**0.21**	**0.22**	0.40
Vegetables (times/week)	8.14 (5.51)	**−0.23**	**0.72**	−0.03	7.85 (5.17)	−0.08	**0.73**	−0.06	0.39
Fruit (times/week)	7.94 (5.04)	−0.09	**0.77**	0.07	8.15(4.99)	−0.01	**0.71**	−0.02	0.50
SASBs (times/week)	5.71 (4.92)	**0.43**	0.07	**0.20**	6.90 (5.98)	**0.62**	0.09	0.09	**<0.001**
Sweet snacks & desserts (times/week)	10.44 (5.90)	**0.66**	−0.06	0.05	10.98 (6.30)	**0.64**	−0.04	0.09	0.16
Processed meat (times/week)	0.99 (1.20)	**0.57**	0.02	0.11	1.15 (1.38)	**0.56**	0.13	−0.04	0.07
French fries (times/week)	0.95 (0.99)	**0.73**	−0.02	0.01	1.06 (0.99)	**0.50**	−0.02	0.15	0.07
Crisps (times/week)	0.81 (0.86)	**0.67**	−0.06	−0.07	0.79(0.86)	**0.55**	−0.07	−0.17	0.86
Screen time (hours/day)	1.26 (0.83)	**0.27**	**−0.23**	**0.53**	1.37(0.80)	**0.52**	**−0.34**	**0.25**	**0.03**
Outdoor play (standardized by season)	−0.13 (0.94)	−0.03	0.11	**0.68**	0.07(1.05)	0.00	−0.11	**0.73**	**0.002**
Walking (hours/day)	0.69 (0.45)	−0.07	0.09	**0.79**	0.73(0.51)	0.12	−0.01	**0.77**	0.12
Sleep (hours/day)	10.89 (0.46)	−0.15	0.09	**−0.30**	10.86(0.49)	**−0.37**	0.14	**0.34**	0.23
% variance explained		17.8	12.8	10.6		17.2	11.8	10.6	
Component label (i.e., lifestyle pattern label)		Discretionary Consumption, Low Vegetables, High Screen	Fish, Dairy products, Fruit & Vegetables, Low Screen	SASBs, High Screen, Outdoor Play, Walking, Low Sleep		Discretionary Consumption, High Screen, Low Sleep	Fish, Dairy products, Fruit & Vegetables, Low Screen	Dairy products, High Screen, Outdoor Play, Walking, High Sleep	

Factor loadings represent the correlations of all standardized EBRBs with a given component (i.e., lifestyle pattern). In bold: Factor loadings >0.20 or <−0.20 and *p* value < 0.05. *p* values were obtained from *t*-test comparing mean values between girls and boys. PCA: Principal component analysis; SD: Standard deviation; SASBs: Sugar or artificially sweetened beverages.

**Table 3:** Betas (95% CI) from multiply imputed and weighted hierarchical multivariable linear regression analyses stratified by sex, with unhealthy lifestyle patterns as the dependent variables. The EDEN study.

	Girls (n = 459) Discretionary Consumption, Low Vegetables, High Screen		Boys (n = 519) Discretionary Consumption, High Screen, Low Sleep	
	β (95% CI)	*p*	β (95% CI)	*p*
***MODEL 1: FAMILY SOCIO-DEMOGRAPHY***				
**Maternal education level at inclusion**		0.16		**0.007**
<HS diploma vs. ≥3-y university degree	0.25 (−0.07; 0.58)		0.33 (0.04; 0.62)	
≥HS diploma to 2-y university degree vs. ≥3-y university degree	0.02 (−0.23; 0.28)		−0.04 (−0.27; 0.20)	
**Paternal education level at inclusion**		**0.024**		**0.031**
<HS diploma vs. ≥3-y university degree	0.35 (0.04; 0.66)		0.27 (−0.02; 0.57)	
≥HS diploma to 2-y university degree vs. ≥3-y university degree	0.08 (−0.19; 0.36)		0.03 (−0.23; 0.29)	
**Monthly household income**		0.66		0.16
2300 EUR vs. 3800 EUR	−0.09 (−0.41; 0.23)		0.29 (−0.01; 0.60)	
2301 EUR to 3800 EUR vs. 3800 EUR	0.03 (−0.21; 0.28)		0.17 (−0.07; 0.42)	
**Perceived financial hardship ^1^**				
At least one vs. No				
**Maternal employment status**		0.33		0.60
Not employed vs. Employed full-time	0.18 (−0.09; 0.45)		0.07 (−0.17; 0.32)	
Employed part-time vs. Employed full-time	0.12 (−0.10; 0.34)		−0.06 (−0.27; 0.15)	
**Study center**				
Nancy vs. Poitiers			−0.09 (−0.27; 0.09)	0.33
**Maternal age at delivery**				0.10
<27 y vs. >33 y			0.10 (−0.14; 0.35)	
27 to 33 y vs. >33 y			−0.12 (−0.34; 0.09)	
**Mother lives alone (no other adults)**				
Yes vs. No				
**Younger siblings at home**				
At least one vs. No				
**Older siblings at home**				
At least one vs. No				
**Pets at home**		**0.050**		0.06
At least one dog vs. No pets	0.29 (0.05; 0.52)		0.25 (0.03; 0.47)	
No dog but other animals vs. No pets	0.07 (−0.15; 0.29)		0.19 (−0.02; 0.40)	
**Backyard at home**				
Yes vs. No	−0.44 (−0.76; −0.12)	**0.007**		
***MODEL 2 ^2^: PARENTS’ HEALTH/BEHAVIORS***				
**Mother’s diet during pregnancy (PCA scores)**				
Healthy			0.15 (0.05; 0.24)	**0.002**
Western	0.38 (0.22; 0.54)	**<0.001**	0.40 (0.30; 0.49)	**<0.001**
Mother’s leisure time physical activity		0.70		0.67
0 h/week vs. >2 h/week	0.09 (−0.21; 0.39)		0.09 (−0.15; 0.33)	
>0 to 2 h/week vs. >2 h/week	0.01 (−0.30; 0.33)		0.03 (−0.23; 0.28)	
**Father’s leisure time physical activity**		0.41		**0.012**
0 h/week vs. >2 h/week	0.03 (−0.19; 0.26)		0.20 (0.01; 0.40)	
>0 to 2 h/week vs. >2 h/week	0.16 (−0.12; 0.44)		−0.08 (−0.32; 0.16)	
**Mother’s BMI**		0.24		
>30 kg/m^2^ vs. <25 kg/m^2^	0.18 (−0.07; 0.44)			
25 to 30 kg/m^2^ vs. <25 kg/m^2^	0.07 (−0.15; 0.30)			
**Mother’s depression symptoms (CESD score)**	0.00 (−0.01; 0.01)	0.45	0.01 (−0.01; 0.02)	0.27
**Smoking status**				
At least one parent vs. Neither parent	−0.03 (−0.22; 0.16)	0.74	0.08 (−0.09; 0.26)	0.35
**Childcare arrangements outside school**				0.57
OSHC services vs. Mother, father, or family			−0.01 (−0.20; 0.18)	
Neighbor or employee vs. parents or family			−0.11 (−0.34; 0.12)	
**Daily canteen meals**				
Yes vs. No				
***MODEL 3 ^2^: PARENT-CHILD INTERACTIONS***				
**Parental feeding practices at year 2**, (T3 vs. T1 + T2)				
**Child control (Lack of parental control)**	0.19 (−0.01; 0.40)	0.06	0.05 (−0.13; 0.22)	0.60
**Food as reward**	−0.05 (−0.24; 0.14)	0.63	0.04 (−0.12; 0.21)	0.62
**Food restrictions for health**	−0.11 (−0.29; 0.07)	0.22	−0.08 (−0.24; 0.08)	0.31
**Pressure to eat**				
**Daily breakfast intake**				
Yes vs. No				
**TV on during meals**		**0.003**		**<0.001**
Often or always vs. Never	0.37 (0.13; 0.61)		0.46 (0.26; 0.65)	
Sometimes vs. Never	0.31 (0.10; 0.53)		0.38 (0.20; 0.56)	
**Unhealthy snacking outside meals**		**<0.001**		**<0.001**
Often or always vs. Never	0.69 (0.40; 0.99)		0.57 (0.33; 0.81)	
Sometimes vs. Never	0.03 (−0.18; 0.24)		0.04 (−0.14; 0.23)	
**SASBs during meals**				
Yes vs. No	−0.11 (−0.35; 0.13)	0.39	0.48 (0.30; 0.67)	**<0.001**
**Parents’ perception of child physical activity**				
More active than vs. Less or as active as other children				
**Participation in an organized sports activity**				
Yes vs. No	−0.12 (−0.30; 0.06)	0.19	−0.09 (−0.24; 0.06)	0.23
**Bedtime (Hours)**	0.24 (0.03; 0.45)	**0.024**	0.30 (0.14; 0.46)	**<0.001**
**Cognitive stimulation (HOME score)**	−0.02 (−0.05; 0.02)	0.42		
**Activities with the father (PCA scores)**				
Everyday care with leisure time				
Everyday care without active leisure time			−0.04 (−0.12; 0.03)	0.26

If not otherwise stated, variables were collected at year 5. In bold: *p* value < 0.05. BMI: Body mass index; CES-D: Centre for Epidemiologic Studies-Depression; CI: Confidence interval; HOME: Home observation measurement of the environment; HS: High school; OSHC: Outside school hours care; PCA: Principal component analysis; SASBs: Sugar or artificially sweetened beverages; T: Tertile; y: years. ^1^ If the “monthly household income” variable was retained in the univariable analysis, the “perceived financial hardship” variable was automatically not retained. ^2^ For the sake of parsimony, the effect of each variable was adjusted for the variables from the same block and those from the preceding block(s) when *p* < 0.20.

**Table 4:** Betas (95% CI) from multiply imputed and weighted hierarchical multivariable linear regression analyses, stratified by sex with healthy lifestyle pattern as the dependent variables. The EDEN study.

	Girls (n = 459) Fish, Dairy Products, Fruit & Vegetables, Low Screen		Boys (n = 519) Fish, Dairy Products, Fruit & Vegetables, Low Screen	
	β (95% CI)	*p*	β (95% CI)	*p*
***MODEL 1: FAMILY SOCIO-DEMOGRAPHY***				
**Maternal education level at inclusion**		0.10		0.20
<HS diploma vs. ≥3-y university degree	−0.22 (−0.52; 0.09)		−0.27 (−0.56; 0.03)	
≥HS diploma to 2-y university degree vs. ≥3-y university degree	0.05 (−0.20; 0.30)		−0.15 (−0.39; 0.09)	
**Paternal education level at inclusion**		0.51		0.06
<HS diploma vs. ≥3-y university degree	−0.17 (−0.48; 0.14)		−0.32 (−0.63; −0.02)	
≥HS diploma to 2-y university degree vs. ≥3-y university degree	−0.14 (−0.41; 0.13)		−0.27 (−0.53; 0.00)	
**Monthly household income**		0.08		0.69
2300 EUR vs. 3800 EUR	−0.29 (−0.61; 0.03)		−0.08 (−0.41; 0.25)	
2301 EUR to 3800 EUR vs. 3800 EUR	−0.26 (−0.50; −0.02)		−0.10 (−0.35; 0.15)	
**Perceived financial hardship ^1^**				
At least one vs. No				
**Maternal employment status**				**0.034**
Not employed vs. Employed full-time			0.31 (0.02; 0.60)	
Employed part-time vs. Employed full-time			0.17 (−0.06; 0.39)	
**Study center**				
Nancy vs. Poitiers				
**Maternal age at delivery**				
<27 y vs. >33 y				
27 to 33 y vs. > 33 y				
**Mother lives alone (no other adults)**				
Yes vs. No	0.46 (0.09; 0.83)	**0.014**	−0.15 (−0.51; 0.22)	0.43
**Younger siblings at home**				
At least one vs. No			0.09 (−0.10; 0.28)	0.34
**Older siblings at home**				
At least one vs. No	0.15 (−0.03; 0.33)	0.10		
**Pets at home**				
At least one dog vs. No pets				
No dog but other animals vs. No pets				
**Backyard at home**				
Yes vs. No				
***MODEL 2 ^2^: PARENTS’ HEALTH/BEHAVIORS***				
**Mother’s diet during pregnancy (PCA scores)**				
Healthy	0.28 (0.17; 0.39)	**<0.001**	0.28 (0.18; 0.38)	**<0.001**
Western	−0.11 (−0.22; 0.01)	0.06	−0.05 (−0.15; 0.04)	0.26
**Mother’s leisure time physical activity**		0.53		0.29
0 h/week vs. >2 h/week	−0.17 (−0.47; 0.11)		−0.20 (−0.45; 0.06)	
>0 to 2 h/week vs. >2 h/week	−0.14 (−0.45; 0.17)		−0.15 (−0.43; 0.13)	
**Father’s leisure time physical activity**				
0 h/week vs. >2 h/week				
>0 to 2 h/week vs. >2 h/week				
**Mother’s BMI**		0.70		
>30 kg/m^2^ vs. <25 kg/m^2^	−0.01 (−0.27; 0.24)			
25 to 30 kg/m^2^ vs. <25 kg/m^2^	−0.09 (−0.30; 0.13)			
**Mother’s depression symptoms (CESD score)**	−0.01 (−0.02; 0.00)	**0.046**		
**Smoking status**				
At least one parent vs. Neither parent	−0.17 (−0.36; 0.02)	0.09	0.03 (−0.16; 0.21)	0.78
**Childcare arrangements outside school**				
OSHC services vs. Mother, father, or family				
Neighbor or employee vs. parents or family				
**Daily canteen meals**				
Yes vs. No			−0.07 (−0.25; 0.12)	0.47
***MODEL 3 ^2^: PARENT-CHILD INTERACTIONS***				
**Parental feeding practices at year 2**, (T3 vs. T1 + T2)				
**Child control (Lack of parental control)**	−0.08 (−0.29; 0.13)	0.47	−0.04 (−0.24; 0.16)	0.68
**Food as reward**	−0.08 (−0.28; 0.12)	0.42	−0.14 (−0.34; 0.05)	0.15
**Food restrictions for health**	0.13 (−0.08; 0.34)	0.24	0.20 (0.01; 0.38)	**0.035**
**Pressure to eat**				
**Daily breakfast intake**				
Yes vs. No	0.16 (−0.15; 0.46)	0.32		
**TV on during meals**		**<0.001**		**0.005**
Often or always vs. Never	−0.30 (−0.55; −0.06)		−0.19 (−0.42; 0.04)	
Sometimes vs. Never	0.22 (0.01; 0.44)		0.08 (−0.13; 0.30)	
**Unhealthy snacking outside meals**		0.25		0.13
Often or always vs. Never	−0.12 (−0.40; 0.17)		−0.11 (−0.40; 0.17)	
Sometimes vs. Never	−0.18 (−0.38; 0.04)		−0.21 (−0.43; 0.00)	
**SASBs during meals**				
Yes vs. No	−0.21 (−0.46; 0.04)	0.10	0.03 (−0.18; 0.25)	0.76
**Parents’ perception of child physical activity**				
More active than vs. Less or as active as other children			−0.21 (−0.41; −0.01)	**0.042**
**Participation in an organized sports activity**				
Yes vs. No	0.04 (−0.14; 0.22)	0.67	0.23 (0.05; 0.40)	**0.01**
**Bedtime (Hours)**	−0.13 (−0.35; 0.08)	0.23	−0.36 (−0.54; −0.17)	**<0.001**
**Cognitive stimulation (HOME score)**	0.07 (0.03; 0.11)	**0.001**	0.06 (0.02; 0.10)	**0.001**
**Activities with the father (PCA scores)**				
Everyday care with leisure time				
Everyday care without active leisure time			0.06 (−0.03; 0.15)	0.20

If not otherwise stated, variables were collected at year 5. In bold: *p* value < 0.05. BMI: Body mass index; CES-D: Centre for Epidemiologic Studies-Depression; CI: Confidence interval; HOME: Home observation measurement of the environment; HS: High School; OSHC: Outside school hours care; PCA: Principal component analysis; SASBs: Sugar or artificially sweetened beverages; T: Tertile; y: years. ^1^ If the “monthly household income” variable was retained in the univariable analysis, the "perceived financial hardship" variable was automatically not retained. ^2^ For the sake of parsimony, the effect of each variable was adjusted for the variables from the same block and those from the preceding block(s) when *p* < 0.20.

**Table 5:** Betas (95% CI) from multiply imputed and weighted hierarchical multivariable linear regression analyses, stratified by sex with mixed lifestyle pattern as the dependent variables. The EDEN study.

	Girls (n = 459) SASBs, High Screen, Outdoor Play, Walking, Low Sleep		Boys (n = 519) Dairy Products, High Screen, Outdoor Play, Walking, High Sleep	
	β (95% CI)	*p*	β (95% CI)	*p*
***MODEL 1: FAMILY SOCIO-DEMOGRAPHY***				
**Maternal education level at inclusion**		**<0.001**		**0.004**
<HS diploma vs. ≥3-y university degree	0.71 (0.41; 1.01)		0.39 (0.11; 0.68)	
≥HS diploma to 2-y university degree vs. 3-y university degree	0.15 (−0.09; 0.39)		0.02 (−0.20; 0.25)	
**Paternal education level at inclusion**		0.27		**0.016**
<HS diploma vs. ≥3-y university degree	0.21 (−0.09; 0.50)		0.39 (0.10; 0.67)	
≥HS diploma to 2-y university degree vs. ≥3-y university degree	0.07 (−0.20; 0.35)		0.19 (−0.06; 0.44)	
**Monthly household income**		0.07		0.47
2300 EUR vs. 3800 EUR	0.35 (0.05; 0.65)		0.18 (−0.11; 0.47)	
2301 EUR to 3800 EUR vs. 3800 EUR	0.19 (−0.04; 0.43)		0.08 (−0.15; 0.32)	
**Perceived financial hardship ^1^**				
At least one vs. No				
**Maternal** **employment status**		0.13		0.17
Not employed vs. Employed full-time	−0.22 (−0.49; 0.04)		0.02 (−0.22; 0.26)	
Employed part-time vs. Employed full-time	−0.18 (−0.38; 0.03)		−0.17 (−0.37; 0.03)	
**Study center**				
Nancy vs. Poitiers			0.26 (0.09; 0.44)	**0.003**
**Maternal age at delivery**				
<27y vs. >33 y				
27 to 33 y vs. >33 y				
**Mother lives alone (no other adults)**				
Yes vs. No				
**Younger siblings at home**				
At least one vs. No	−0.05 (−0.26; 0.15)	0.61		
**Older siblings at home**				
At least one vs. No	0.21 (0.02; 0.41)	**0.033**	0.17 (−0.01; 0.34)	0.06
**Pets at home**		**0.003**		**0.011**
At least one dog vs. No pets	0.31 (0.09; 0.53)		0.25 (0.04; 0.46)	
No dog but other animals vs. No pets	−0.05 (−0.26; 0.16)		−0.06 (−0.27; 0.14)	
**Backyard at home**				
Yes vs. No				
***MODEL 2 ^2^: PARENTS HEALTH/BEHAVIORS***				
**Mother’s diet during pregnancy (PCA scores)**				
Healthy				
Western	0.00 (−0.11; 0.12)	0.96	0.01 (−0.08; 0.10)	0.86
**Mother’s leisure time physical activity**		0.78		**<0.001**
0 h/week vs. >2 h/week	0.08 (−0.22; 0.37)		−0.47 (−0.72; −0.23)	
>0 to 2 h/week vs. >2 h/week	0.11 (−0.20; 0.42)		−0.42 (−0.68; −0.16)	
**Father’s leisure time physical activity**		0.40		**0.017**
0 h/week vs. >2 h/week	0.06 (−0.16; 0.29)		−0.19 (−0.39; 0.00)	
>0 to 2 h/week vs. >2 h/week	−0.11 (−0.39; 0.17)		−0.32 (−0.56; −0.08)	
**Mother’s BMI**		0.22		0.13
>30 kg/m^2^ vs. <25 kg/m^2^	0.17 (−0.08; 0.42)		0.17 (−0.07; 0.42)	
25 to 30 kg/m^2^ vs. <25 kg/m^2^	0.16 (−0.05; 0.38)		0.18 (−0.02; 0.39)	
**Mother’s depression symptoms (CESD score)**	0.00 (−0.01; 0.01)	0.50	0.00 (−0.01; 0.01)	0.62
**Smoking status**				
At least one parent vs. Neither parent	0.07 (−0.12; 0.26)	0.46	0.07 (−0.11; 0.24)	0.43
**Childcare arrangements outside school**		0.25		**<0.001**
OSHC services vs. Mother, father, or family	−0.19 (−0.42; 0.04)		−0.39 (−0.60; −0.19)	
Neighbor or employee vs. parents or family	−0.06 (−0.31; 0.18)		−0.24 (−0.48; 0.01)	
**Daily canteen meals**				
Yes vs. No			−0.12 (−0.32; 0.08)	0.23
***MODEL 3 ^2^: PARENT-CHILD INTERACTIONS***				
**Parental feeding practices at year 2**, (T3 vs. T1 + T2)				
**Child control (Lack of parental control)**				
**Food as reward**	0.10 (−0.10; 0.29)	0.33		
**Food restrictions for health**				
**Pressure to eat**				
**Daily breakfast intake**				
Yes vs. No	−0.46 (−0.77; −0.14)	**0.005**		
**TV on during meals**		0.44		0.86
Often or always vs. Never	0.14 (−0.10; 0.38)		0.04 (−0.18; 0.26)	
Sometimes vs. Never	0.01 (−0.20; 0.23)		0.06 (−0.15; 0.26)	
**Unhealthy snacking outside meals**		0.09		0.20
Often or always vs. Never	−0.06 (−0.34; 0.22)		0.23 (−0.04; 0.51)	
Sometimes vs. Never	0.17 (−0.04; 0.38)		0.16 (−0.05; 0.36)	
**SASBs during meals**				
Yes vs. No	0.08 (−0.17; 0.33)	0.53	0.16 (−0.04; 0.37)	0.12
**Parents’ perception of child physical activity**				
More active than vs. Less or as active as other children			0.12 (−0.08; 0.31)	0.24
**Participation in an organized sports activity**				
Yes vs. No	−0.07 (−0.25; 0.10)	0.42		
**Bedtime (Hours)**	0.40 (0.19; 0.61)	**<0.001**	−0.51 (−0.69; −0.32)	**<0.001**
**Cognitive stimulation (HOME score)**			−0.01 (−0.05; 0.03)	0.61
**Activities with the father (PCA scores)**				
Everyday care with leisure time				
Everyday care without active leisure time	−0.08 (−0.18; 0.01)	0.08	−0.07 (−0.15; 0.01)	0.11

If not otherwise stated, variables were collected at year 5. In bold: *p* value < 0.05. BMI: Body mass index; CES-D: Centre for Epidemiologic Studies-Depression; CI: Confidence interval; HOME: Home observation measurement of the environment; HS: High school; OSHC: Outside school hours care; PCA: Principal component analysis; SASBs: Sugar or artificially sweetened beverages; T: Tertile; y: years. ^1^ If the “monthly household income” variable was retained in the univariable analysis, the “perceived financial hardship” variable was automatically not retained. ^2^ For the sake of parsimony, the effect of each variable was adjusted for the variables from the same block and those from the preceding block(s) when *p* < 0.20.

## Data Availability

All data supporting the findings of this study are included in the present article or the present article or supplemental material.

## Supplementary Materials

The following are available online at https://www.mdpi.com/article/10.3390/nu13113803/s1, Additional file 1. Additional Text S1: Definition of family socioecological factors. Additional Text S2: Handling missing data—detailed procedure. Additional Table S1: Distribution of father-child activities and PCA factor loadings for patterns of father’s involvement in child rearing (n= 1081). The EDEN study. Additional Table S2: Proportions of missing data and imputation models used for contextual factors of interest (for respondents at year 5, n = 978). The EDEN study. Additional Table S3: Odds ratios (95% CIs) from multiply imputed logistic regression analyses, with having (or not) all EBRB data at year 5 (and thus considered a respondent at year 5, n = 978) as the dependent variable. The EDEN study. Additional Table S4: Characteristics of included and excluded participants. The EDEN study. Additional Table S5: Betas (95% CI) from multiply imputed and weighted hierarchical univariable linear regression analyses, stratified by sex with unhealthy lifestyle pattern as the dependent variable. The EDEN study. Additional Table S6: Betas (95% CI) from multiply imputed and weighted hierarchical univariable linear regression analyses, stratified by sex with healthy lifestyle pattern as the dependent variable. The EDEN study. Additional Table S7: Betas (95% CI) from multiply imputed and weighted hierarchical univariable linear regression analyses, stratified by sex with mixed lifestyle pattern as the dependent variable. The EDEN study.
