# Supplementary material for: Family Socioecological Correlates of Lifestyle Patterns in Early Childhood: A Cross-Sectional Study from the EDEN Mother–Child Cohort
Source: Nutrients. 2021 Oct 26;13(11):3803. doi: 10.3390/nu13113803 (PMC8623755; doi:10.3390/nu13113803)
Supplement: Supplementary file 1 [file nutrients-13-03803-s001.zip › nutrients-1382983-supplementary.pdf]

Family socioecological correlates of lifestyle patterns in early childhood: a cross-sectional study from the EDEN mother-child cohort.

Additional Text S1. Definition of family socioecological factors.

We selected 35 contextual factors and organized them in three nested blocks, as described below. These were collected 5-year post-partum, except as otherwise stated.

Family socio-demography (block 1) - *Socioeconomic position*: maternal and paternal education levels at inclusion referred to the highest diploma obtained (<high school diploma; high school diploma to 2-year university degree; ≥3-year university degree), monthly household income (<2,300 euros; 2,300 to 3,800 euros; >3,800 euros), perceived financial hardship (at least one difficulty ; no difficulty in the past year to purchase goods for the family among the followings: food, clothes, accommodation related bills, or medical care/drugs), maternal employment status (not employed; employed part-time; employed full time).

Socio-demographic factors: study center (Poitiers; Nancy), mother lives alone (without other adult) (yes; no), maternal age at delivery (<27 years; 27 to 33 years; >33 years).

Family structure: older sibling(s) at home (at least one older sibling; no older siblings), younger sibling(s) at home (at least one younger sibling; no younger siblings), pet(s) at home (at least one dog; no dog but another pet(s); no pet), backyard at home (yes; no).

Parents' health/behaviors (block 2) – *Parents' choices for external care*: daily canteen meals (yes; no), childcare arrangements: outside school hours care (OSHC) services or by an employee, neighbors or friends, or by parents or family). In this study, OSHC services are defined as programs for children before and after school and on school holidays. OSHC programs take place predominantly in the school environment.

Parents' behaviors: mother's and father's leisure-time physical activity (0 hour/week; >0 to 2 hours/week; >2 hours/week); and scores on two dietary patterns (labelled as Healthy and Western) identified in mothers during pregnancy in a previous analysis [1].

Parents' health: smoking status (at least one smoking parent; no smoking parent) and mother's BMI (<25 kg/m^2; 25 to 30 kg/m^2; >30 kg/m^2). We also used the Centre for Epidemiologic Studies-Depression Scale [CES-D] to evaluate mothers' depressive symptoms (continuous score) [2].

Parent-child interactions (block 3): TV on during meals (often or always; never; sometimes); "unhealthy" snacking outside meals (often or always; never; sometimes); SASBs during meals (yes; no); daily breakfast intake (yes; no); four parental feeding practices at year 2 (child control; food as reward; restriction for health; pressure to eat), assessed with the Child Feeding Practices Questionnaire (CFPQ) [3,4] and categorized according to the tertiles of their distribution); child's participation in an organized sports activity (yes; no); parents' perception of his/her child physical activity (more active than other children; less active than or as active as other children); bedtime (in hours); and cognitive stimulation, assessed as the sum of three subscores of the Home Observation Measurement of the Environment (HOME) [5], i.e., language stimulation, academic stimulation, and variety of experience.

Finally, fathers were asked how often they did each of the following 7 activities with their child (times/week, ranging from "every day or almost" to "never or almost never"): bathing, putting to bed, singing, reading a story, playing games at home, taking him/her for a walk, and doing active leisure time with him/her. This information was synthesized into two patterns of activities using PCA (without Varimax rotation) on the standardized variables (**Additional Table 1**). The first component was characterized by high levels of all seven activities and was therefore labeled "everyday care with leisure time". The second component, labeled "everyday care without active leisure time", was defined by high levels of bathing, putting to bed, and reading a story, along with low levels of shared active time, i.e., playing games at home, taking the

child for a walk, or doing physical activity with her/him. The higher the score, the more the father adhered to the component (and vice versa).

Additional Text S2. Handling missing data – detailed procedure.

In the sample we analyzed of 978 children, 4 of the 35 contextual variables studied had no missing data (study center, maternal age at delivery, bedtime, and childcare arrangements outside school). Of the remaining 31 variables, missing data rates ranged from 0.1% for the variables of “mother lives alone”, “pets at home” and “TV on during meals” to 12.8% for the “cognitive stimulation” variable (**Additional Table S2**).

Main analysis – IPW/MI procedure. Inspired by Caldwell et al. [6], we first imputed the lifestyle pattern scores, the contextual factors, and the potential predictors of response at year 5 for all 1,907 children with a known birth date, by using the fully conditional specification approach. However, the sample we analyzed comprised the 978 children with all EBRB data (and therefore with scores for all three lifestyle patterns identified) (**Figure 2**). On the assumption that data were missing at random, 20 independent datasets were generated. The imputation models included all variables of interest after they were ranked in ascending order of missing data. Categorical variables were imputed with multinomial models, binary variables with logistic regressions, and continuous variables with predictive mean matching (**Additional Table S2**).

Then we derived stabilized weights from logistic regression models by using variables associated with response (i.e., having complete data for all EBRBs under study and therefore scores for all lifestyle patterns) at year 5 ($P<0.20$), including the child's birth weight, the study center, maternal and paternal education levels, household monthly income at baseline, mother lives alone at baseline, smoking before pregnancy, primiparity, perceived financial hardship at baseline, and maternal age at delivery (**Additional Table S3**). We weighted the child included in the analysis at age 5 by the inverse of the probability of responding to all EBRBs of interest at year 5 and used the Hosmer-Lemeshow test to assess model fit [7,8]. To further assess the IPW building process we checked the following suggested requirements: we did not observe problems with large weights and we confirmed a high degree of overlap between weights among participants who did and did not have scores for each identified lifestyle pattern [7,8].

We combined each inverse probability weighting effect estimator and its corresponding sandwich variance estimator according to Rubin’s Rules [9]. To obtain a global *P*-value for categorical variables, we used the median *P*-value of the significance tests conducted in each imputed dataset [10].

Sensitivity analyses. To appreciate the relevance of this methodology for dealing with missing data, we conducted sensitivity analyses with three different approaches. In the first analysis, called Complete Cases (CC)/Multiple Imputation (MI), we included the 978 children with available EBRBs data (and therefore scores for all lifestyle patterns) and imputed data for contextual factors, without redressing this sample of respondents. In the second analysis, called MI/MI, we analyzed the 1,907 children with a known birth date and imputed data for both the outcomes (i.e., scores for all lifestyle patterns) and the contextual factors. Finally, we analyzed the cases that would be considered complete in the strict sense of the term (CC/CC), i.e., the 487 children with complete data for both behavioral and contextual factors (**Figure 2**). In view of the multistage nature of our multivariable analysis, the variables used for these three sensitivity analyses were those selected from the main analysis so that any differences could be attributed to the approach.

Additional Table S1. Distribution of father-child activities and PCA factor loadings for patterns of fathers' involvement in child rearing (n= 1081). The EDEN study.

	Distribution of father-child activities	PCA factor loadings	
	Mean (SD)	Component 1	Component 2
Bathing (times/week)	2.38 (2.33)	**0.59**	**0.43**
Putting to bed (times/week)	4.10 (2.67)	**0.58**	**0.55**
Singing (times/week)	1.41 (2.01)	**0.61**	-0.03
Reading a story (times/week)	1.99 (2.21)	**0.62**	**0.43**
Playing games at home (times/week)	2.36 (2.17)	**0.72**	**-0.23**
Taking him/her for a walk (times/week)	1.65 (1.58)	**0.67**	**-0.47**
Doing physical activity with him/her (times/week)	1.51 (1.58)	**0.69**	**-0.50**
% explained variance		41.6	17.1
Component label		Everyday care with leisure time	Everyday care without physical leisure time

In bold: Factor loadings >0.20 or < -0.20.

PCA: Principal component analysis; SD: Standard deviation.

Additional Table S2. Proportion of missing data and imputation models used for contextual factors of interest (for respondents at year 5, n=978). The EDEN study.

Variables	Type	Imputation model	% missing
**Family (Block 1)**			
Maternal education level at inclusion	Categorical	Multinomial	1.3
Paternal education level at inclusion	Categorical	Multinomial	5.8
Monthly household income	Categorical	Multinomial	2.8
Perceived financial hardship	Dichotomous	Logistic	0.8
Maternal working status	Categorical	Multinomial	0.8
Study center	Dichotomous	No missing data	0
Maternal age at delivery	Categorical	No missing data	0
Mother lives alone	Dichotomous	Logistic	0.1
Younger sibling(s) at home	Dichotomous	Logistic	1.9
Older sibling(s) at home	Dichotomous	Logistic	0.4
Pet(s) at home	Categorical	Multinomial	0.1
Backyard at home	Dichotomous	Logistic	5.7
**Parents (Block 2)**			
Mother's diet during pregnancy (PCA scores)			
Healthy	Continuous	Predictive mean matching	11.5
Western	Continuous	Predictive mean matching	11.5
Mother's leisure-time physical activity	Categorical	Multinomial	1.9
Father's leisure-time physical activity	Categorical	Multinomial	9.9
Mother's BMI	Categorical	Multinomial	1.1
Mother's depressive symptoms (CES-D score)	Dichotomous	Logistic	1.3
Smoking status	Dichotomous	Logistic	4.1
Childcare arrangements outside school	Categorical	No missing data	0
Daily canteen meals	Dichotomous	Logistic	5.3
**Parent-child interactions (block 3)**			
Parental feeding practices at year 2			
Child control	Dichotomous	Logistic	9.8
Food as a reward	Dichotomous	Logistic	8.9
Restriction for health	Dichotomous	Logistic	9.8
Pressure to eat	Dichotomous	Logistic	9.8
Daily breakfast intake	Dichotomous	Logistic	0.6
TV on during meals	Categorical	Multinomial	0.1
Unhealthy snacking outside meals	Categorical	Multinomial	0.4
SASBs during meals	Dichotomous	Logistic	0.2
Parents' perception of child's activity level	Dichotomous	Logistic	0.5
Participation in organized sports activity	Dichotomous	Logistic	0.4
Bedtime (Hours)	Continuous	No missing data	0
Cognitive stimulation (HOME score)	Continuous	Predictive mean matching	12.8
Activities with the father (PCA scores)			
Everyday care with leisure time	Quantitative	Predictive mean matching	9.9
Everyday care without physical leisure time	Quantitative	Predictive mean matching	9.9

If not otherwise stated, variables were collected at year 5.

BMI: Body mass index; CES-D: Centre for Epidemiologic Studies-Depression; HOME: Home observation measurement of the environment; PCA: Principal component analysis; SASBs: Sugar or artificially sweetened beverages

Additional Table S3. Odds ratios (95% CIs) from multiply imputed logistic regression analyses, with having (or not) all EBRB data at year 5 (and thus considered a respondent at year 5, n=978) as the dependent variable. The EDEN study.

Variables	OR (95% CI)	*P*
**Study center**		
Nancy vs. Poitiers	0.88 (0.74; 1.06)	**0.17**
**Maternal education level at inclusion**		**<0.0001**
≤High school diploma vs. ≥3-y university degree	0.30 (0.24; 0.39)	
2-y university degree vs. ≥3-y university degree	0.61 (0.49; 0.76)	
**Paternal education level at inclusion**		**<0.0001**
≤High school diploma vs. ≥3-y university degree	0.42 (0.33; 0.54)	
2-y university degree vs. ≥3-y university degree	0.68 (0.53; 0.87)	
**Household monthly income at inclusion**		**<0.0001**
<€ 2,300 vs. >€ 3,800	0.46 (0.37; 0.58)	
€ 2,301 to 3 800 vs. >€ 3,800	0.82 (0.63; 1.05)	
**Perceived financial hardship at inclusion**		
At least one vs. None during the past year	0.49 (0.33; 0.72)	**0.0003**
**Mother lives alone at inclusion**		
Yes vs. No	0.33 (0.18; 0.59)	**0.0002**
**Primiparity at inclusion**		
Yes vs. No	1.28 (1.07; 1.54)	**0.007**
**Smoking before pregnancy**		**<0.0001**
0 cigarette vs. ≥10 cigarettes/day	2.02 (1.62; 2.52)	
<10 vs. ≥10 cigarettes/day	1.00 (0.73; 1.37)	
**Mother's BMI before pregnancy**		0.98
<25 vs. >30 kg/m^2	0.97 (0.70; 1.34)	
25 to 30 vs. >30 kg/m^2	1.00 (0.79; 1.27)	
**Maternal age at delivery**		**<0.0001**
<27 y vs. >33 y	0.45 (0.35; 0.58)	
27 to 33 y vs. >33 y	0.74 (0.59; 0.92)	
**Child sex**		
Boys vs. Girls	0.96 (0.80; 1.15)	0.64
**Child birth weight (grams)**	1.00 (1.00; 1.00)	**0.0008**
**Socioeconomic and psychosocial adversity at year 3**		
Yes vs. No	0.90 (0.68; 1.17)	0.42

BMI: Body mass index; CI: Confidence interval; OR: Odds ratio; y: years

Additional Table S4. Characteristics of included and excluded participants. The EDEN study.

Characteristics	Included n[1]=978	Excluded n[1]=929	*P*
**Maternal education level at inclusion**			**<0.0001**
≤High school diploma	192 (19.9)	348 (38.3)	
High school diploma to 2-y university degree	392 (40.6)	354 (38.9)	
≥3-y university degree	381 (39.5)	207 (22.8)	
**Paternal education level at inclusion**			**<0.0001**
≤High school diploma	277 (30.1)	341 (43.6)	
High school diploma to 2-y university degree	372 (40.4)	293 (37.5)	
≥3-y university degree	272 (29.5)	148 (18.9)	
**Household monthly income at inclusion**			**<0.0001**
<€ 2,300	374 (38.5)	507 (54.9)	
€ 2,301 to 3,800	282 (29.0)	217 (23.5)	
>€ 3,800	316 (32.5)	199 (21.6)	
**Perceived financial hardship at inclusion**			
At least one during the past year	40 (4.2)	73 (8.25)	**0.0003**
**Mother lives alone at inclusion**			
Yes	16 (1.6)	45 (4.9)	**<0.0001**
**Primiparity at inclusion**			
Yes	464 (47.5)	383 (41.4)	**0.0067**
**Smoking before pregnancy**			**<0.0001**
0 cigarette	691 (71.6)	510 (55.5)	
<10 cigarettes/day	99 (10.3)	148 (16.1)	
≥10 cigarettes/day	175 (18.1)	261 (28.4)	
**Mother's BMI before pregnancy**			0.95
<25 kg/m^2	710 (73.7)	665 (73.5)	
25 to 30 kg/m^2	170 (17.6)	159 (17.6)	
>30 kg/m^2	82 (8.7)	81 (8.9)	
**Maternal age at delivery**			**<0.0001**
<27 y	214 (21.9)	315 (33.9)	
27 to 33 y	475 (48.6)	424 (45.6)	
>33 y	289 (29.6)	190 (20.4)	
**Child' sex**			0.64
Boys	439 (46.9)	444 (48.0)	
**Child birth weight (grams)**	3242.9 (427)	3170.5 (430.3)	**0.0004**

Values are means (SD) or *n* (%). Chi-square and Student t tests were used to compare frequencies and means, respectively. **In bold**: *P* value <0.05.

BMI: Body mass index; y: years

[1] n max

Additional Table S5. Betas (95% CI) from multiply imputed and weighted hierarchical univariable linear regression analyses, stratified by sex with unhealthy lifestyle pattern as the dependent variable. The EDEN study.

	Girls (n=459)		Boys (n=519)	
	Discretionary Consumption, Low Vegetables, High Screen		Discretionary Consumption, High Screen, Low sleep	
	β (95% CI)	*P*-value	β (95% CI)	*P*-value
Block 1: Family				
**Mother's education level at inclusion**		**<0.0001**		**<0.0001**
< High school diploma vs. ≥ 3-y university degree	0.53 (0.29; 0.78)		0.76 (0.54; 0.98)	
< HS diploma to 2-y university degree vs. ≥ 3-y university degree	0.16 (-0.06; 0.39)		0.21 (0.00; 0.42)	
**Father's education level at inclusion**		**<0.0001**		**<0.0001**
< HS diploma vs. ≥ 3-y university degree	0.51 (0.26; 0.75)		0.68 (0.44; 0.93)	
< HS diploma to 2-y university degree vs. ≥ 3-y university degree	0.15 (-0.09; 0.39)		0.26 (0.01; 0.50)	
**Monthly household income**		**0.0155**		**<0.0001**
€2,300 vs. € 3,800	0.39 (0.12; 0.65)		0.72 (0.48; 0.96)	
€2,301 to 3,800 vs. € 3,800	0.20 (-0.03; 0.42)		0.37 (0.15; 0.59)	
**Perceived financial hardship**				
At least one vs. No	0.50 (0.25; 0.75)	**<0.0001**	0.48 (0.24; 0.71)	**<0.0001**
**Maternal employment status**		**0.0259**		**0.0061**
Not employed vs. Employed full-time	0.34 (0.09; 0.59)		0.34 (0.11; 0.57)	
Employed part-time vs. Employed full-time	0.15 (-0.07; 0.37)		-0.03 (-0.24; 0.19)	
**Study center** [2,3]				
Nancy vs. Poitiers	0.09 (-0.10; 0.28)	0.34	-0.19 (-0.37; -0.01)	**0.0440**
**Maternal age at delivery** [1,2]		0.72		**0.0014**
<27 y vs. >33 y	0.08 (-0.19; 0.35)		0.29 (0.04; 0.54)	
27 to 33 y vs. >33 y	-0.01 (-0.25; 0.22)		-0.11 (-0.33; 0.11)	
**Mother lives alone (no other adult)** [1,2]				
Yes vs. No	0.06 (-0.30; 0.42)	0.74	0.17 (-0.17; 0.51)	0.33
**Younger sibling(s) at home**				
At least one vs. No	-0.04 (-0.23; 0.15)	0.67	0.03 (-0.15; 0.22)	0.73
**Older sibling(s) at home**				
At least one vs. No	0.08 (-0.11; 0.27)	0.43	0.10 (-0.09; 0.28)	0.31
**Pet(s) at home** [1]		**0.0047**		**0.0011**
At least one dog vs. No pets	0.38 (0.15; 0.61)		0.41 (0.19; 0.62)	
No dog but other animals vs. No pets	0.09 (-0.13; 0.32)		0.25 (0.03; 0.47)	
**Backyard at home**				
Yes vs. No	-0.44 (-0.76; -0.12)	**0.0064**	-0.17 (-0.49; 0.16)	0.31
Block 2: Parents				
**Mother's diet during pregnancy (PCA scores)**				
Healthy	0.11 (-0.13; 0.35)	0.36	0.08 (-0.03; 0.18)	**0.15**
Western	0.43 (0.28; 0.58)	**<.0001**	0.48 (0.40; 0.57)	**<0.0001**
**Mother's leisure time physical activity**		**0.0071**		**0.0003**
0 hour/week vs. >2 hours/week	0.35 (0.05; 0.65)		0.36 (0.10; 0.62)	
>0 to 2 hours/week vs. >2 hours/week	0.06 (-0.27; 0.39)		-0.03 (-0.31; 0.26)	
**Father's leisure time physical activity**		**0.11**		**<0.0001**
0 hour/week vs. >2 hours/week	0.24 (0.01; 0.46)		0.44 (0.21; 0.67)	
>0 to 2 hours/week vs. >2 hours/week	0.14 (-0.17; 0.44)		-0.01 (-0.29; 0.28)	
**Mother's BMI**		**0.14**		0.26
>30 kg/m^2 vs. <25 kg/m^2	0.25 (-0.02; 0.51)		0.20 (-0.07; 0.48)	
25 to 30 kg/m^2 vs. <25 kg/m^2	0.14 (-0.09; 0.37)		0.07 (-0.16; 0.29)	
**Mother's depressive symptoms (CES-D score)**	0.01 (0.00; 0.02)	**0.0239**	0.02 (0.01; 0.03)	**0.0008**
**Smoking status**				
At least one parent vs. Neither parent	0.20 (0.00; 0.39)	**0.0449**	0.37 (0.19; 0.56)	**<0.0001**
**Childcare arrangements outside school** [1,2]		0.22		**0.0057**
Outside school hours care services vs. Mother, father, or family	-0.16 (-0.39; 0.06)		-0.23 (-0.43; -0.03)	
Neighbor or employee vs. Mother, father, or family	0.03 (-0.20; 0.27)		-0.38 (-0.63; -0.13)	
**Daily canteen meals**				
Yes vs. No	-0.12 (-0.31; 0.08)	0.23	0.04 (-0.14; 0.23)	0.64
Block 3: Parent-child interactions				
**Parental feeding practices at year 2,** (T3 vs. T1 + T2)				
**Child control (Lack of parental control)**	0.27 (0.04; 0.50)	**0.0203**	0.33 (0.10; 0.56)	**0.0046**
**Food as reward**	0.15 (-0.07; 0.36)	**0.18**	0.27 (0.04; 0.49)	**0.0207**
**Food restrictions for health**	-0.23 (-0.44; -0.02)	**0.0322**	-0.23 (-0.45; -0.02)	**0.0316**
**Pressure to eat**	0.06 (-0.16; 0.28)	0.61	0.00 (-0.25; 0.26)	0.98
**Daily breakfast intake** [2]				
Yes vs. No	0.05 (-0.29; 0.39)	0.78	-0.07 (-0.41; 0.28)	0.71
**TV on during meals** [1]		**<0.0001**		**<0.0001**

Often or always vs. Never	0.77 (0.55; 1.00)		0.97 (0.76; 1.17)	
Sometimes vs. Never	0.36 (0.13; 0.58)		0.60 (0.39; 0.81)	
**Unhealthy snacking outside meals**		**<0.0001**		**<0.0001**
Often or always vs. Never	1.20 (0.93; 1.47)		1.20 (0.94; 1.47)	
Sometimes vs. Never	0.26 (0.05; 0.47)		0.33 (0.12; 0.55)	
**SASBs during meals** [1]				
Yes vs. No	0.43 (0.18; 0.67)	**<0.0001**	1.00 (0.79; 1.20)	**<0.0001**
**Parents' perception of child physical activity**				
More active vs. Less active or as active as other children	-0.10 (-0.39; 0.20)	0.52	-0.01 (-0.23; 0.20)	0.91
**Participation in an organized sports activity** [1]				
Yes vs. No	-0.26 (-0.45; -0.07)	**0.0089**	-0.29 (-0.47; -0.10)	**0.0020**
**Bedtime (Hours)** [1,2]	0.62 (0.41; 0.83)	**<0.0001**	0.64 (0.45; 0.83)	**<0.0001**
**Cognitive stimulation (HOME score)**	-0.04 (-0.08; 0.00)	**0.0701**	-0.01 (-0.06; 0.03)	0.4912
**Activities with the father (PCA scores)**				
Everyday care with leisure time	-0.06 (-0.16; 0.04)	0.26	-0.07 (-0.17; 0.04)	0.21
Everyday care without physical leisure time	-0.03 (-0.14; 0.08)	0.61	-0.18 (-0.28; -0.09)	**0.0003**

If not otherwise stated, variables were collected at year 5.

In bold: *P*<0.20.

BMI: Body mass index; CES-D: Centre for Epidemiologic Studies-Depression; CI: Confidence interval; HOME: Home observation measurement of the environment; HS: High school; PCA: Principal component analysis; SASBs: Sugar or artificially sweetened beverages; T: Tertile; y: years

[1,2]analyses performed respectively on girls and boys with complete data

Additional Table S6. Betas (95% CI) from multiply imputed and weighted hierarchical univariable linear regression analyses, stratified by sex with healthy lifestyle pattern as the dependent variable. The EDEN study.

	Girls (n=459)		Boys (n=519)	
	Fruits, Vegetables, Dairy, Fish, Less Screen		Fruits, Vegetables, Dairy, Fish, Less Screen	
	β (95% CI)	*P*-value	β (95% CI)	*P*-value
Block 1: Family				
**Mother's education level at inclusion**		**0.0044**		**0.0024**
< High school diploma vs. ≥ 3-y university degree	-0.39 (-0.63; -0.15)		-0.38 (-0.61; -0.15)	
< HS diploma to 2-y university degree vs. ≥ 3-y university degree	-0.11 (-0.33; 0.11)		-0.28 (-0.50; -0.07)	
**Father's education level at inclusion**		**0.0137**		**0.0001**
< HS diploma vs. ≥ 3-y university degree	-0.35 (-0.60; -0.10)		-0.5 (-0.74; -0.25)	
< HS diploma to 2y university degree vs. ≥ 3-y university degree	-0.24 (-0.48; 0.00)		-0.39 (-0.63; -0.15)	
**Monthly household income**		**0.0057**		**0.0211**
€2,300 vs. € 3,800	-0.36 (-0.61; -0.11)		-0.30 (-0.54; -0.06)	
€2,301 to 3,800 vs. € 3,800	-0.32 (-0.54; -0.10)		-0.28 (-0.51; -0.06)	
**Perceived financial hardship**				
At least one vs. No	-0.16 (-0.40; 0.09)	0.21	-0.03 (-0.26; 0.20)	0.81
**Maternal employment status**		0.99		**0.10**
Not employed vs. Employed full-time	0.01 (-0.24; 0.25)		0.21 (-0.03; 0.46)	
Employed part-time vs. Employed full-time	0.01 (-0.20; 0.22)		0.16 (-0.06; 0.38)	
**Study center**[2,3]				
Nancy vs. Poitiers	-0.08 (-0.27; 0.10)	0.39	0.08 (-0.10; 0.26)	0.39
**Maternal age at delivery** [1,2]		0.37		0.83
<27 y vs. >33 y	-0.19 (-0.45; 0.07)		-0.07 (-0.32; 0.18)	
27 to 33 y vs. >33 y	-0.10 (-0.32; 0.13)		-0.01 (-0.23; 0.21)	
**Mother lives alone** [1,2]				
Yes vs. No	0.33 (-0.02; 0.67)	**0.06**	-0.24 (-0.58; 0.09)	**0.16**
**Younger sibling(s) at home**				
At least one vs. No	-0.02 (-0.21; 0.16)	0.81	0.19 (0.01; 0.37)	**0.0379**
**Older sibling(s) at home**				
At least one vs. No	0.13 (-0.05; 0.32)	**0.16**	-0.05 (-0.23; 0.13)	0.56
**Pet(s) at home** [1]		0.53		0.22
At least one dog vs. No pets	0.01 (-0.22; 0.23)		-0.19 (-0.41; 0.02)	
No dog but other animals vs. No pets	-0.11 (-0.33; 0.11)		-0.08 (-0.30; 0.14)	
**Backyard at home**				
Yes vs. No	-0.10 (-0.41; 0.21)	0.52	0.14 (-0.18; 0.46)	0.39
Block 2: Parents				
**Mother's diet during pregnancy (PCA scores)**				
Healthy	0.30 (0.19; 0.42)	**<.0001**	0.32 (0.22; 0.41)	**<.0001**
Western	-0.17 (-0.28; -0.06)	**0.0020**	-0.10 (-0.18; -0.01)	**0.0375**
**Mother's leisure time physical activity**		**0.0045**		**0.0257**
0 hour/week vs. >2 hours/week	-0.46 (-0.75; -0.17)		-0.36 (-0.61; -0.10)	
>0 to 2 hours/week vs. >2 hours/week	-0.26 (-0.58; 0.06)		-0.25 (-0.53; 0.04)	
**Father's leisure time physical activity**		0.59		0.34
0 hour/week vs. >2 hours/week	-0.11 (-0.34; 0.11)		0.04 (-0.17; 0.26)	
>0 to 2 hours/week vs. >2 hours/week	-0.10 (-0.39; 0.19)		0.18 (-0.09; 0.45)	
**Mother's BMI**		**0.19**		0.25
>30 kg/m^2 vs. <25 kg/m^2	-0.06 (-0.32; 0.19)		-0.20 (-0.48; 0.07)	
25 to 30 kg/m^2 vs. <25 kg/m^2	-0.20 (-0.43; 0.02)		-0.13 (-0.36; 0.09)	
**Mother's depressive symptoms (CES-D score)**	-0.01 (-0.02; 0 .00)	**0.0260**	0.00 (-0.01; 0.01)	0.92
**Smoking status**				
At least one parent vs. Neither parent	-0.25 (-0.43; -0.06)	**0.0081**	-0.12 (-0.31; 0.07)	**0.19**
**Childcare arrangements outside school** [1,2]		0.91		0.21
Outside school hours care services vs. Mother, father, or family	0.04 (-0.17; 0.26)		0.05 (-0.15; 0.25)	
Neighbor or employee vs. Mother, father, or family	0.00 (-0.23; 0.23)		0.22 (-0.03; 0.46)	
**Daily canteen meals**				
Yes vs. No	0.05 (-0.14; 0.24)	0.59	-0.12 (-0.30; 0.06)	**0.19**
Block 3: Parent-child interactions				
**Parental feeding practices at year 2,** (T3 vs. T1 + T2)				
**Child control (Lack of parental control)**	-0.17 (-0.39; 0.06)	**0.14**	-0.18 (-0.40; 0.05)	**0.12**
**Food as reward**	-0.25 (-0.46; -0.04)	**0.0199**	-0.28 (-0.49; -0.06)	**0.0129**
**Food restrictions for health**	0.23 (0.01; 0.45)	**0.0439**	0.27 (0.07; 0.46)	**0.0071**
**Pressure to eat**	-0.05 (-0.26; 0.16)	0.62	0.09 (-0.11; 0.30)	0.38
**Daily breakfast intake** [2]				
Yes vs. No	0.35 (0.02; 0.68)	**0.0363**	0.20 (-0.14; 0.54)	0.24
**TV on during meals** [1]		**<.0001**		**<.0001**

Often or always vs. Never	-0.60 (-0.81; -0.38)		-0.48 (-0.70; -0.27)	
Sometimes vs. Never	0.14 (-0.07; 0.35)		0.01 (-0.21; 0.22)	
**Unhealthy snacking outside meals**		**0.0047**		**0.0001**
Often or always vs. Never	-0.47 (-0.75; -0.19)		-0.50 (-0.77; -0.22)	
Sometimes vs. Never	-0.18 (-0.40; 0.04)		-0.46 (-0.69; -0.24)	
**SASBs during meals** [1]				
Yes vs. No	-0.46 (-0.70; -0.22)	**0.0001**	-0.14 (-0.36; 0.07)	**0.19**
**Parents' perception of child's physical activity**				
More active vs. Less active than or as active as other children	0.05 (-0.23; 0.33)	0.72	-0.26 (-0.47; -0.05)	**0.0158**
**Participation in an organized sports activity** [1]				
Yes vs. No	0.17 (-0.02; 0.35)	**0.08**	0.35 (0.18; 0.53)	**<.0001**
**Bedtime (Hours)** [1,2]	-0.34 (-0.55; -0.13)	**0.0014**	-0.53 (-0.71; -0.34)	**<.0001**
**Cognitive stimulation (HOME score)**	0.06 (0.02; 0.10)	**0.0043**	0.08 (0.04; 0.12)	**<.0001**
**Activities with the father (PCA scores)**				
Everyday care with leisure time	0.05 (-0.04; 0.15)	0.27	0.06 (-0.05; 0.16)	0.28
Everyday care without physical leisure time	-0.02 (-0.12; 0.09)	0.77	0.10 (0.00; 0.20)	**0.0468**

If not otherwise stated, variables were collected at year 5.

In bold: P<0.20.

BMI: Body mass index; CES-D: Centre for Epidemiologic Studies-Depression; CI: Confidence interval; HOME: Home observation measurement of the environment; HS: High school; PCA: Principal component analysis; SASBs: Sugar or artificially sweetened beverages; T: Tertile; y: years

[1,2] analyses performed respectively on girls and boys with complete data

Additional Table S7. Betas (95% CI) from multiply imputed and weighted hierarchical univariable linear regression analyses, stratified by sex with mixed lifestyle pattern as the dependent variable. The EDEN study.

	Girls (n=459)		Boys (n=519)	
	SASBs, Walking, Outdoor Play, Screen, Less Sleep		Dairy, Walking, Outdoor Play, High Screen, High Sleep	
	β (95% CI)	P-value	β (95% CI)	P-value
Block 1: Family				
**Mother's education level at inclusion**		**<0.0001**		**<0.0001**
< High school diploma vs. ≥ 3-y university degree	0.99 (0.76; 1.23)		0.71 (0.49; 0.93)	
< HS diploma to 2-y university degree vs. ≥ 3-y university degree	0.31 (0.10; 0.52)		0.17 (-0.03; 0.38)	
**Father's education level at inclusion**		**<0.0001**		**<0.0001**
< HS diploma vs. ≥ 3-y university degree	0.74 (0.50; 0.98)		0.67 (0.44; 0.91)	
< HS diploma to 2-y university degree vs. ≥ 3-y university degree	0.38 (0.13; 0.62)		0.29 (0.05; 0.52)	
**Monthly household income**		**<0.0001**		**<0.0001**
€2,300 vs. € 3,800	0.79 (0.54; 1.05)		0.58 (0.34; 0.81)	
€2,301 to 3,800 vs. € 3,800	0.38 (0.16; 0.60)		0.22 (0,00; 0.43)	
**Perceived financial hardship**				
At least one vs. No	0.20 (-0.05; 0.45)	**0.11**	0.35 (0.13; 0.58)	**0.0022**
**Maternal employment status**		**0.10**		**0.0011**
Not employed vs. Employed full-time	0.23 (-0.02; 0.48)		0.33 (0.11; 0.56)	
Employed part-time vs. Employed full-time	-0.05 (-0.26; 0.17)		-0.12 (-0.32; 0.09)	
**Study center** [2,3]				
Nancy vs. Poitiers	-0.11 (-0.30; 0.08)	0.26	0.15 (-0.03; 0.33)	**0.10**
**Maternal age at delivery** [1,2]		0.29		0.32
<27 y vs. >33 y	-0.20 (-0.47; 0.06)		0.08 (-0.16; 0.32)	
27 to 33 y vs. >33 y	-0.16 (-0.39; 0.08)		-0.08 (-0.30; 0.13)	
**Mother lives alone** [1,2]				
Yes vs. No	-0.09 (-0.45; 0.26)	0.61	0.18 (-0.15; 0.51)	0.29
**Younger sibling(s) at home**				
At least one vs. No	-0.23 (-0.42; -0.04)	**0.0201**	-0.1 (-0.28; 0.08)	0.27
**Older sibling(s) at home**				
At least one vs. No	0.30 (0.11; 0.49)	**0.0022**	0.26 (0.09; 0.44)	**0.0036**
**Pet(s) at home** [1]		**<0.0001**		**0.0018**
At least one dog vs. No pets	0.54 (0.31; 0.77)		0.35 (0.14; 0.56)	
No dog but other animals vs. No pets	0.04 (-0.18; 0.26)		0.01 (-0.20; 0.22)	
**Backyard at home**				
Yes vs. No	-0.10 (-0.41; 0.21)	0.52	-0.01 (-0.33; 0.32)	0.96
Block 2: Parents				
**Mother's diet during pregnancy (PCA scores)**				
Healthy	0.03 (-0.08; 0.14)	0.58	0.03 (-0.07; 0.14)	0.52
Western	0.14 (0.02; 0.26)	**0.0198**	0.09 (-0.01; 0.18)	**0.08**
**Mother's leisure time physical activity**		**0.0054**		**0.0002**
0 hour/week vs. >2 hours/week	0.37 (0.07; 0.67)		-0.39 (-0.64; -0.15)	
>0 to 2 hours/week vs. >2 hours/week	0.09 (-0.24; 0.42)		-0.59 (-0.86; -0.32)	
**Father's leisure time physical activity**		**0.0005**		**0.0157**
0 hour/week vs. >2 hours/week	0.28 (0.05; 0.50)		-0.16 (-0.37; 0.05)	
>0 to 2 hours/week vs. >2 hours/week	-0.19 (-0.48; 0.10)		-0.36 (-0.63; -0.10)	
**Mother's BMI**		**0.0001**		**0.0064**
>30 kg/m^2 vs. <25 kg/m^2	0.42 (0.16; 0.68)		0.30 (0.04; 0.55)	
25 to 30 kg/m^2 vs. <25 kg/m^2	0.41 (0.19; 0.64)		0.30 (0.08; 0.51)	
**Mother's depressive symptoms (CES-D score)**	0.01 (0.00; 0.02)	**0.0345**	0.01 (0.00; 0.03)	**0.0211**
**Smoking status**				
At least one parent vs. Neither parent	0.27 (0.08; 0.46)	**0.0062**	0.19 (0.01; 0.37)	**0.0376**
**Childcare arrangements outside school** [1,2]		**0.0014**		**<.0001**
Outside school hours care services vs. Mother, father, or family	-0.41 (-0.63; -0.19)		-0.58 (-0.77; -0.39)	
Neighbor or employee vs. Mother, father, or family	-0.23 (-0.46; 0.01)		-0.37 (-0.60; -0.13)	
**Daily canteen meals**				
Yes vs. No	-0.04 (-0.24; 0.16)	0.68	-0.34 (-0.51; -0.16)	**0.0002**
Block 3: Parent-child interactions				
**Parental feeding practices at year 2** (T3 vs. T1 + T2)				
**Child control (Lack of parental control)**	-0.08 (-0.32; 0.17)	0.54	-0.10 (-0.31; 0.11)	0.36
**Food as reward**	0.24 (0.02; 0.47)	**0.0324**	-0.05 (-0.25; 0.15)	0.62
**Food restrictions for health**	-0.01 (-0.25; 0.23)	0.93	-0.02 (-0.21; 0.18)	0.87
**Pressure to eat**	0.10 (-0.14; 0.35)	0.40	-0.07 (-0.27; 0.13)	0.50
**Daily breakfast intake** [2]				
Yes vs. No	-0.76 (-1.09; -0.42)	**<0.0001**	-0.05 (-0.39; 0.28)	0.75
**TV on during meals** [1]		**<0.0001**		**0.0040**

Often or always vs. Never	0.62 (0.39; 0.84)		0.36 (0.15; 0.57)	
Sometimes vs. Never	0.23 (0.01; 0.46)		0.21 (-0.01; 0.42)	
**Unhealthy snacking outside meals**		**0.0020**		**0.0028**
Often or always vs. Never	0.48 (0.19; 0.77)		0.48 (0.21; 0.76)	
Sometimes vs. Never	0.34 (0.11; 0.56)		0.22 (0.00; 0.44)	
**SASBs during meals** [1]				
Yes vs. No	0.56 (0.32; 0.80)	**<0.0001**	0.39 (0.18; 0.60)	**0.0003**
**Parents' perception of child's physical activity**				
More active vs. Less active than or as active as other children	0.16 (-0.13; 0.45)	0.29	0.24 (0.03; 0.45)	**0.0226**
**Participation in an organized sports activity** [1]				
Yes vs. No	-0.29 (-0.48; -0.09)	**0.0040**	0.01 (-0.17; 0.18)	0.95
**Bedtime (Hours)** [1,2]	0.70 (0.49; 0.91)	**<0.0001**	-0.30 (-0.49; -0.11)	**0.0017**
**Cognitive stimulation (HOME score)**	-0.01 (-0.06; 0.03)	0.61	-0.04 (-0.08; 0.00)	**0.0402**
**Activities with the father (PCA scores)**				
Everyday care with leisure time	0.03 (-0.06; 0.13)	0.50	0.04 (-0.06; 0.14)	0.43
Everyday care without physical leisure time	-0.17 (-0.27; -0.07)	**0.0012**	-0.16 (-0.25; -0.07)	**0.0008**

If not otherwise stated, variables were collected at year 5.

In bold: P<0.20.

BMI: Body mass index; CES-D: Centre for Epidemiologic Studies-Depression; CI: Confidence interval; HOME: Home observation measurement of the environment; HS: High school; PCA: Principal component analysis; SASBs: Sugar or artificially sweetened beverages; T: Tertile; y: years

[1,2]analyses performed respectively on girls and boys with complete data

Supplemental references:

1. on behalf of the EDEN mother-child cohort study group; Yuan, W.L.; Nicklaus, S.; Lioret, S.; Lange, C.; Forhan, A.; Heude, B.; Charles, M.-A.; de Lauzon-Guillain, B. Early Factors Related to Carbohydrate and Fat Intake at 8 and 12 Months: Results from the EDEN Mother–Child Cohort. *Eur J Clin Nutr* **2017**, *71*, 219–226, doi:10.1038/ejcn.2016.216.
2. Weissman, M.M.; Sholomskas, D.; Pottenger, M.; Prusoff, B.A.; Locke, B.Z. Assessing Depressive Symptoms in Five Psychiatric Populations: A Validation Study. *Am J Epidemiol* **1977**, *106*, 203–214, doi:10.1093/oxfordjournals.aje.a112455.
3. Musher-Eizenman, D.; Holub, S. Comprehensive Feeding Practices Questionnaire: Validation of a New Measure of Parental Feeding Practices. *Journal of Pediatric Psychology* **2007**, *32*, 960–972, doi:10.1093/jpepsy/jsm037.
4. Musher-Eizenman, D.R.; de Lauzon-Guillain, B.; Holub, S.C.; Leporc, E.; Charles, M.A. Child and Parent Characteristics Related to Parental Feeding Practices. A Cross-Cultural Examination in the US and France. *Appetite* **2009**, *52*, 89–95, doi:10.1016/j.appet.2008.08.007.
5. Bradley, R.H.; Caldwell, B.M. Home Observation for Measurement of the Environment: A Revision of the Preschool Scale. *American Journal of Mental Deficiency.* **1979**, *84*, 235–244.
6. Caldwell, T.M.; Rodgers, B.; Clark, C.; Jefferis, B.J.M.H.; Stansfeld, S.A.; Power, C. Lifecourse Socioeconomic Predictors of Midlife Drinking Patterns, Problems and Abstention: Findings from the 1958 British Birth Cohort Study. *Drug and Alcohol Dependence* **2008**, *95*, 269–278, doi:10.1016/j.drugalcdep.2008.01.014.
7. Seaman, S.R.; White, I.R.; Copas, A.J.; Li, L. Combining Multiple Imputation and Inverse-probability Weighting. *Biometrics* **2012**, *68*, 129–137, doi:10.1111/j.1541-0420.2011.01666.x.
8. Seaman, S.R.; White, I.R. Review of Inverse Probability Weighting for Dealing with Missing Data. *Stat Methods Med Res* **2013**, *22*, 278–295, doi:10.1177/0962280210395740.
9. Rubin, D. *Multiple Imputation for Nonresponse in Surveys.*; Wiley.; 1987;
10. Eekhout, I.; van de Wiel, M.A.; Heymans, M.W. Methods for Significance Testing of Categorical Covariates in Logistic Regression Models after Multiple Imputation: Power and Applicability Analysis. *BMC Med Res Methodol* **2017**, *17*, 129, doi:10.1186/s12874-017-0404-7.
